# Constraints on percussive seismic signals in a noisy environment by European fiddler crabs, *Afruca tangeri*

**DOI:** 10.1242/jeb.249323

**Published:** 2025-04-10

**Authors:** Tom Mulder, Yiyuan Yang, Ellen Morley, Thomas E. Miller, Daniel Hending, Graham K. Taylor, Beth Mortimer

**Affiliations:** ^1^ University of Oxford, Department of Biology, 11a Mansfield Road, Oxford OX1 3SZ, UK; ^2^ University of Oxford, Department of Computer Science, 7 Parks Road, Oxford OX1 3QG, UK

**Keywords:** Vibrational communication, Percussion, Biomechanics, Geophones, Courtship

## Abstract

Many animals communicate using seismic vibrations. Signaller morphology, signal production method and environmental factors impose interacting constraints that may be impossible to replicate in the laboratory, making it essential to study seismic communication *in situ*. Here, we focused on the constraints on percussive seismic signals in European fiddler crabs (*Afruca tangeri*), recording a large dataset of percussive seismic signals *in situ*, and testing for waveform differences as a function of signaller morphology and behaviour. In addition, we aimed to characterise signal degradation and interference by seismic noise from wind and vibrated vegetation in the natural environment. We obtained over 8000 percussive seismic signal recordings, and found that although the length, rhythm and loudness of the signals all varied as a function of behaviour, their frequency content did not. Consequently, behaviours could be discriminated based on seismic recordings alone. Larger claws were only associated with louder signals in the case of claw drumming behaviours, but morphology did not affect percussive signal features otherwise. Environmental effects on percussive signals were substantial as signals attenuated significantly over distance, and wind speed was positively correlated with seismic noise, albeit independently of distance to vegetation. We conclude that percussive seismic signals are limited in their ability to convey information through frequency, but that their broadband nature is advantageous in the face of noise and frequency filtering by the substrate. In contrast, changing the amplitude and repetition rate of a percussive signal offers a simple but effective means for small animals to communicate seismically in noisy environments.

## Teaser

**Highlighted Article:** Percussive behaviours of wild fiddler crabs (*Afruca tangeri*) recorded with geophones produce broadband signals that can be categorised by their rhythm and intensity.

## INTRODUCTION

Seismic communication via substrate-borne vibrations is common in animals of all sizes (Hill, 2001; O'Connell-Rodwell et al., 2007), but technological challenges have meant that this remains under-researched *in situ* compared with acoustic communication (Hill et al., 2019). Understanding seismic communication in wild animals is important because many rely on seismic vibrations for day-to-day tasks such as prey/mate detection and discrimination of individuals in groups, despite seismic noise and substrate variations (Hill, 2001; Mortimer et al., 2021; Mulder et al., 2020; O'Connell-Rodwell et al., 2007). Crucially, environmental substrates can drastically impact vibrational signals through attenuation, dispersion and scattering (Arnason et al., 2002; Mortimer et al., 2018) and may be irreplicable in the laboratory.

The production of substrate vibrations (known as ‘seismic vibrations’ when the ground is the vibrated substrate) is varied across the animal kingdom (Hill, 2009). Examples include stridulation, tremulation, tymbal buckling, air–ground coupling of vocalisations, and percussion. In short, vibration generation through stridulation is achieved by rubbing body parts together, whereas tremulation involves shaking (parts of) the body in contact with the substrate without a percussive impact, and tymbal buckling involves the rapid buckling of a stiff tymbal organ (Davranoglou et al., 2019; Hill, 2009, 2008; Hill et al., 2019; Hill and Wessel, 2016). Vocalisations using vocal cords can vibrate the substrate either directly through vibration of the body in contact with the substrate or indirectly via air–ground coupling of the airborne pressure wave (O'Connell-Rodwell et al., 2001). Percussion generally involves direct vibration production by impacting a substrate with (part of) the body (Hill and Wessel, 2016; O'Connell-Rodwell et al., 2001, 2000), but can also be achieved indirectly by striking two body parts together to generate a vibration in the body, which consequently vibrates the substrate. We henceforth refer to ‘indirect percussive vibration production’ where two body parts are struck together to generate vibrations.

Examples include: chameleons vibrating their bodies on leaves (Barnett et al., 1999), African elephants vibrating the ground through low-frequency vocalisations (Mortimer et al., 2018; O'Connell-Rodwell et al., 2001), and golden moles and kangaroo rats vibrating the ground by striking the substrate (Hill, 2009; Mason and Narins, 2002; Takeshita et al., 2018; Takeshita and Murai, 2016). Even apparently rudimentary seismic signals can serve various tasks and differ between species. In kangaroo rats (*Dipodomys* genus), percussive foot stomping signals deter predators, inform neighbours of one's position and minimise territorial conflicts (Randall, 1984; Randall and Boltas King, 2001; Randall and Matocq, 1997). Moreover, although signal patterns (rhythms of foot strikes) can be complex and differ between species in the genus *Dipodomys*, signals are broadband (200–2000 Hz) and do not display intra-signal frequency modulation, and inter-individual signal variation has not been observed (Hill, 2009; Randall, 1984; Randall and Boltas King, 2001).

From a biomechanical perspective, percussion is a simple method of vibration generation (Hill, 2009). Where the seismic vibrations produced by methods such as vocalisation are modulated in loudness and frequency content to convey complex information to distant individuals (Reinwald et al., 2021), percussive impacts may have less scope for complex information content. Simplicity arises because there are limited ways to strike a surface with a body part (force, time of contact, repetition rate), and percussive substrate strikes typically result in broadband signals owing to the impulse nature of the vibration generation. Nonetheless, assessing the extent to which percussive seismic signals can be manipulated by the signaller is essential in understanding the biology of percussive animals. For example, if morphology determines percussive signal features, then dishonest signalling about morphological characteristics may be impossible, with implications for aggressive competition between males and female choice. Despite these potential constraints and behavioural implications, it remains unknown which features of percussive seismic signals generated *in situ* are manipulated between behaviours, and which are determined by body morphology or abiotic environmental effects such as substrate heterogeneity or propagation distance.

The European fiddler crab (*Afruca tangeri*) is suitable for studying percussive seismic communication and its constraints in the wild. This species occupies the southern mudflats of the Iberian Peninsula (20–50 animals m^−2^) and partakes in percussive seismic communication by drumming. During courtship, *A. tangeri* males wave their major claw and reportedly use it to drum the ground to generate vibrations and attract females (Salmon and Atsaides, 1968). Although the visual aspect has been studied extensively, the vibrational aspect of fiddler crab courtship remains under-researched (Aicher and Tautz, 1990; Mowles et al., 2017; Oliveira and Custódio, 1998; Salmon and Atsaides, 1969; Takeshita and Murai, 2016). Interestingly, ground drumming with the major claw is not reported for all fiddler crab species. For example, *Austruca mjoebergi* generates vibrations through stridulation of the major claw without impacting the substrate (Mowles et al., 2017). Aside from drumming with the major claw reported for *A. tangeri*, their high intensity waving behaviour potentially also causes percussive seismic vibrations. Specifically, the claw waving display of *A. tangeri* reportedly involves two steps, escalating from (1) low intensity waving of only the major claw to (2) high intensity waving in which the carapace and claw are rapidly raised and lowered, as females approach (Jordão et al., 2007). Although step 2 could involve ground impact and thus generate percussive seismic vibrations, the link between the non-drumming behaviour-specific seismic signal features has not been made. Furthermore, whether signal features are affected by male morphology has not been tested, despite potential implications for female choice.

Finally, background recordings of seismic noise variations across the intertidal zone remain absent. This is important as wind and vegetation are known interactive drivers of seismic noise in the (comparable) sandy desert landscape (Mason and Narins, 2002), and vegetation is generally restricted to the upper shore (Wolfrath, 1993). *Afruca tangeri* in the Ria Formosa region are reportedly largest near the upper shore vegetation and smallest on barren lower shore (Wolfrath, 1993). Vegetation can provide cover from potential predators, but may also generate higher levels of noise in windy conditions. Proximity to vegetation could hence impede seismic communication by less loud and potentially smaller individuals, and this cover–noise trade-off may drive the reported size distribution of crabs.

Given the lack of *in situ* seismic communication studies and minimal knowledge about percussive seismic signalling, the present study aimed to investigate the seismic components of fiddler crab courtship and test the following hypotheses: (1) the basic features (sum normalised energy, peak amplitude and peak frequency) of seismic behaviour recordings vary between behaviours of *A. tangeri*, and can be used to classify these behaviours using a machine learning model based on seismic spectrograms alone; (2) basic percussive seismic signal features are affected by signaller morphology; and (3) seismic recordings are influenced by abiotic factors: specifically, percussive seismic signals change over distance, and seismic noise levels are affected by wind speed and proximity to vegetation.

## MATERIALS AND METHODS

### Data collection

#### Study site

Recordings were obtained in the Ria Formosa national park in the Algarve region of Portugal (37.160997°, −7.529444°) during May and June 2022. The site is intertidal, with a sandy substrate and vegetation on the upper shore. Crab behaviours were studied at low tide. Crabs were studied on open ground, with vegetation in the area consisting of sparse and short sea grasses and samphire on the middle ground, and tall reeds and shrubs on the higher ground.

#### Behavioural recordings

Behaviours of male *Afruca tangeri* (Eydoux 1835) occupying 50 focal burrows were recorded for 10–30 min per trial. Focal males were generally positioned at the burrow entrance, and occasionally inside the burrow, and commonly retained occupancy of the focal burrow for the full observation period (27/50 trials). However, original focal males could be replaced mid-trial by competing secondary focal males, which could themselves be replaced by a third and fourth focal male in turn. Occupancy was important to note, as in the statistical tests concerning morphology (outlined further below), only behaviours by the original or final focal male were included depending on whether morphological measurements were obtained preceding or following the observation period, respectively. In statistical tests where morphology was not a tested variable, behaviours by all males that occupied each burrow were included in the analysis to maximise the dataset, while the non-independence of recordings obtained in the same burrow was controlled for. We opted not to include both burrow and crab identity as nested independent variables because the majority of analysed burrows were occupied by one focal male. However, we did not explicitly test signal parameter variation for individuals. Between days, the same focal burrow was never used, and independence was assumed for all focal individuals as physical sizes were compared and identical morphologies were never observed.

**Fig. 1.**
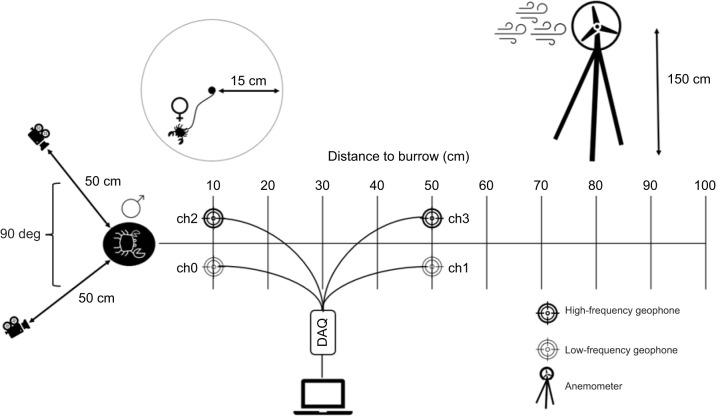
**Data collection overview (not to scale).** Two low- (4.5–160 Hz) and high-frequency (100–400 Hz) geophones were connected to the laptop via the data acquisition unit (DAQ). The geophone pair closest to the burrow was always positioned 10 cm from the burrow entrance. The second pair was moved between 20 and 100 cm from the burrow entrance between trials. Two GoPro cameras recorded (video and audio) the focal male at ∼50 cm from the burrow entrance, at an angle of ∼90 deg to each other with the close low-frequency geophone in view. The anemometer recorded wind speed from atop a 150 cm tall tripod near the experiment location. For some trials, a tethered female was placed near the burrow entrance, attached to a wooden stick by a monofilament line on a plastic sheet with a 15 cm diameter (outlined below). Female and male mass, carapace width and carapace length were measured using a digital scale and callipers, and for males the width and length of the major claw were also measured (Fig. S1).

Two GoPro cameras were placed at ground level within ∼50 cm of the burrow entrance, and were positioned at an ∼90 deg angle to each other, filming at 60 frames s^−1^ and recording audio at a sampling rate of 48 kHz. Concurrent seismic recordings were obtained with two pairs of RT-Clark vertical geophones (Fig. 1). Geophones are among the tools traditionally employed by seismologists and produce reliable recordings owing to tight sensor–substrate coupling via a 3-inch (=7.62 cm) spike vertically inserted into the sand. Firm sensor–substrate contact is essential as inadequate mechanical contact can result in poor vibration detection and noise artifacts caused by factors such as sensor movement relative to the substrate and sensor resonance (Aicher and Tautz, 1990). Wind-shielding of the geophones was decided against because: (1) vertical geophones are minimally sensitive to horizontal forces such as those more likely to be produced by wind, (2) firm placement using the 3-inch spike minimizes wind-induced sensor movements altogether, (3) the shielding device (e.g. an upturned bucket) would likely have been vibrated more severely by the wind, and (4) we aimed to minimise obstruction of the field of view at the burrow entrance. Each pair of geophones consisted of a low-frequency geophone (4.5–160 Hz flat frequency response, 23.4 V m^−1^ s^−1^) and a high-frequency geophone (100–400 Hz flat frequency response, 33.8 V m^−1^ s^−1^), recording at a 1612 Hz sampling frequency. For all trials, one geophone pair was positioned 10 cm from the burrow entrance (‘close pair’). The second pair was positioned at a random distance, 20–100 cm from the burrow entrance at 10 cm intervals (‘far pair’; Fig. 1). Males were generally near the burrow entrance (<20 cm) unless forced out by another male. During body dropping and drumming behaviours specifically (defined in ‘Data analysis’, below), males were always particularly close to their burrow entrance (<10 cm), and burrow distance thus acted as a proxy for distance to signalling focal male. Geophones were connected to LabView (a recording software) via a 24-bit National Instruments Data Acquisition Unit (DAQ). To synchronise video and seismic recordings, the close low-frequency geophone (Fig. 1) was tapped once with the index finger to cause a major spike in the seismic recording. Frame-by-frame video assessment identified impact on the geophone, which was synchronised with the seismic spike.

#### Tethering females to induce rarer male behaviours

After 5 days of observations, it became apparent that rarer behaviours (e.g. high intensity waving and underground drumming) were more common in the presence of females. To induce more instances of these behaviours, one tethered female per trial was placed near the burrow entrance for the final 10–20 min of each trial where possible (*n*♀=25). Females carrying eggs were excluded. Females were tethered to a wooden stick using 10–15 cm of monofilament line glued to the carapace (as per Darden et al., 2019) and placed on a 1.5 mm thick plastic sheet with a 15 cm radius to avoid burrowing (Fig. 1). The sheet was covered with sand, and the stick was pierced through the middle into the sand below. Tethered components of the trials were limited to 20 min. During the preparation phase, females were placed into a natural flowing body of water. Materials were removed from the females' carapace at the end of each trial and no females were injured.

#### Morphological measurements

The mass, major claw width and length, carapace width and carapace length (Fig. S1) of males occupying the focal burrows were measured before or after each trial. Where possible, males were captured using specialised traps (Darden et al., 2019) after trials, but otherwise males were dug up and replaced into another burrow post- measuring. To establish whether crab morphology correlated with the relative vertical position of crabs up the shoreline, morphometric measurements were obtained for a further 72 randomly selected individuals (*n*♂=52, *n*♀=20). These crabs were selected from 14 randomly positioned transects by drawing interval lines perpendicular to the vegetation and waterline at 50 cm intervals, and then randomly selecting one burrow between interval lines from which a crab was retrieved.

#### Environmental measurements

Through 30 trials, wind speed (m s^−1^) was measured using a digital anemometer placed perpendicular to the wind direction atop a 1.5 m tall tripod, at the start of each trial. The height of and proximity to the nearest vegetation from the close geophone pair (Fig. 1) were measured to correlate seismic environmental noise with wind speed and vegetation variables.

### Data analysis

#### Behavioural catalogue

Eight male crab behaviours were temporally tagged in the videos using Behavioural Observation Research Interactive Software (BORIS; Friard and Gamba, 2016), including: (1) waving, (2) simultaneous waves and body drops (simWBD), (3) sequential waves and body drops (seqW+seqBD), (4) underground drumming (UGD), (5) aboveground drumming (AD), (6) locomotion, (7) mud balling (MB) and (8) conflict. Please refer to Table S1 for an extensive ethogram describing these behaviours. To avoid confusion between the claw motion described for aboveground drumming and the claw motions of body dropping behaviours, we urge the readers to view Movies 1 and 2. It should further be noted that aboveground and underground drumming may not be the same bodily motions, as the underground variation was not visually observed.

Point event behaviours (behaviours 1–3, where seqW+seqBD were considered two separate point behaviours) were marked by a singular time stamp, whereas state behaviours that occurred over a stretch of time (behaviours 4−8) were marked by a ‘start’ and ‘stop’ time (Table S1). State behaviours were non-exclusive. For example, a crab could wave whilst locomoting. All behaviours were tagged based on visual inspection of the video recordings, except underground drumming, which was aurally detected from the audio in the video recordings where a knocking sound was heard after a male entered its burrow.

Where a body drop immediately followed a wave, the two movements were defined as two new point behaviours part of the sequential wave and body drop behaviour (i.e. seqW+seqBD; Table S1), distinct from the waving and simultaneous waving and body dropping behaviours. We opted to denote sequential waves and body drops as distinct behavioural events because >1200 body drops were video recorded, and all were closely preceded by waves. Specifically, sequential body drops occurred 0.001–9.4 s following the sequential wave events, where 75% occurred within 1.5 s of the preceding sequential wave, and only six sequential body drop events occurred >4.5 s following the preceding sequential waves.

On occasion, non-focal individuals from neighbouring burrows caused interference in the seismic behaviour recordings. Such instances were removed from the seismic behaviour catalogue by visual assessment of the videos and seismic time signatures. Specifically, the number of observed spikes and assumption of decay over space were used to filter out the erroneous recordings (see Supplementary Materials and Methods). The rate of visually detected erroneous seismic behaviour recordings was 3.8% (*n*_excluded_=311, *n*_retained_=8207). Although this manual approach was unlikely to remove all erroneous seismic recordings from the behaviour catalogue, it ensured exclusion of the most extreme examples.

#### Behavioural grouping

Based on inspection of the log energy boxplots (see Results) and/or body movements observed to produce the vibrations, the individual behaviours were also parsed into three behavioural groups: (1) non-percussive, (2) body dropping and (3) drumming. This grouping approach aimed to emphasize major seismic differences between non-percussive behaviours that exhibit minimal seismic signatures, and two categories of percussive behaviours that appear to generate distinct seismic signatures through different means of percussive vibration production.

The non-percussive group consists of conflict, locomotion, mudballing, waving and sequential waves, and was marked by minimal seismic signals.

The body dropping group consists of simultaneous waves and body drops and sequential body drops, which both produced notable seismic signals and were biomechanically comparable because the carapace was used to percuss the substrate in both cases.

The drumming group consists of the aboveground drumming and underground drumming behaviours. Although the underground behaviour could not be visually compared with the aboveground variant, their seismic signatures were both marked by high repetition rate spikes.

#### Seismic and morphological data analysis

Seismic recordings were parsed per the time stamps from BORIS, and sections of seismic recordings that captured a particular behaviour were collated. The parsed sections included a time buffer to capture the onset and termination of the behaviour such that all point behaviour recordings were kept as point ±0.2 s, whilst state behaviours were filtered to the middle 0.4 s of the signal. Using a 0.4 s window ensured that the signal of separate point behaviour events (e.g. seqW and seqBD, or multiple simWBD events) did not overlap. Underground drumming had a larger buffer before and after the full signal than other behaviours (0.6 versus 0.2 s; Table S1), as its precise onset was more difficult to accurately establish aurally.

To establish whether seismic signal features differ between *A. tangeri* behaviours (hypothesis 1), four basic features were extracted from recordings by the close geophone pair (Ch1 and Ch2 in Fig. 1): peak absolute amplitudes of low- (4.5–160 Hz) and high-frequency (100–400 Hz) geophones, peak frequency for the high- and low-frequency geophones combined (4.5–400 Hz), and the summed normalised energy of the high- and low-frequency geophones combined. Absolute peak amplitudes for each channel were obtained directly from the recordings. Normalised energy for each channel was calculated as normalised energy=sum(velocity amplitude^2^)×1/2 of the 0.4 s recording. To obtain peak frequency, a fast Fourier transform (FFT) was first performed independently on each channel. The FFT of the close high-frequency geophone (Ch2, Fig. 1) was subsequently filtered to >160 Hz and then merged with the low-frequency FFT (4.5–160 Hz) to yield a continuous FFT from 4.5 to 400 Hz. Peak frequency was extracted from this continuous FFT.

To test the effect of male morphological features on peak frequency and loudness of behaviours (hypothesis 2), the recordings were filtered to only retain recordings associated with measured males (*n*♂=41). Three morphological features were analysed: carapace size (Car.1×Car.2 in Fig. S1A), claw size (Claw.1×Claw.2 in Fig. S1B) and mass. All three variables were strongly correlated (claw and carapace size: 0.83; carapace size and mass: 0.89; claw size and mass: 0.94). Only claw size and carapace size were selected for further analyses to assess the effect of the sexually dimorphic trait and general size on signal features, respectively. It should be noted that a principal component analysis (PCA) approach was also considered. However, given the high correlation values, we determined that the simplicity of using the original variables outweighed the potential benefits of PCA in explaining additional variance.

To test whether seismic background noise levels are affected by environmental variables (hypothesis 3), wind speed and distance to vegetation were correlated with seismic energy levels during periods with minimal/no seismic input by crabs. Specifically, we assessed wind speed and vegetation effects on noise levels during waving behaviours (waving and SeqW; see Results). Upon inspection of an energy boxplot, four extreme high energy outliers were observed for SeqW. From the associated spectrograms it was apparent that these events captured the start of the associated body drop (SeqBD), and these outliers were removed from the dataset prior to the noise analyses. Energy levels were calculated as the sum of normalised energy of the close geophone pair, whereas wind speed was calculated as the mean wind speed recorded from 3 s before to 3 s after the waving behaviour.

#### Behavioural classification using residual network classifier

A residual network (ResNet) machine learning (ML) model was used to automatically classify behaviours based on two sets of spectrogram images of the seismic behaviour recordings (hypothesis 1). Fixed length spectrograms [length=0.4 s, window length (WL)=128 or 256, window name=Hamming, Fourier transform overlap=98%] were created for the close low- (4.5–160 Hz) and high-frequency (100–400 Hz) geophone recordings. The 0.4 s signal length was as the same signal sections were analysed as used in the preceding section. Notably, this signal length was set to avoid capturing multiple distinct behaviours (e.g. two distinct simultaneous wave and body drop events, or a sequential wave and body drop).

Although window length ranging from 128 to 1024 samples were initially considered, only the spectrograms with 128- and 256-sample windows were ultimately analysed. Given a sampling frequency of 1612 Hz, a 0.4 s signal equals ∼645 samples. As such, a window length of 1024 samples (=0.64 s) loses all temporal information in the spectrogram and requires zero padding. A window length of 512 equals 0.32 s and likewise causes the loss of important temporal information because Δ*T* (the time difference between consecutive drums) is <0.3 s and a window of 0.32 s would always span multiple drums. To avoid loss of all temporal information of individual drums (mean Δ*T*∼0.16 s, see Results; see Fig. 4), spectrograms with window lengths of 128 (=0.08 s) and 256 (=0.16 s) samples were used for classification using the ResNet ML models.

All spectrograms were pre-processed by removing the white borders, resizing the spectral images to 224×224 pixels, and increasing the sample sizes of rarer behaviours through image duplication to ensure approximately equal sample sizes across behaviours or behavioural groups. Automated classification by the ML algorithms was subsequently performed for the nine individually described behaviours (Table S1), and the three associated behavioural groups.

Three ML simulations were performed for each of the two window lengths, using three pre-processed datasets: (1) low-frequency geophone spectrograms only, (2) high-frequency geophone spectrograms only and (3) a combination of the high- and low-frequency geophone spectrograms. The same classification task was performed for all three datasets of each window length with two different ML pipelines (individual behaviour and behavioural group). For the separate low- and high-frequency spectral images (simulations 1 and 2), a pre-trained ResNet18 network structure was used to mine the features of the spectrogram, and a multi-layer perceptron (including linear, batch normalization and dropout layers) was used to classify the mined features (see Supplementary Materials and Methods, ‘Machine learning’). For simulation 3, including both low- and high-frequency spectrograms, the same pre-trained structure for feature extraction was applied to the separate images, and their features were subsequently concatenated for each classification. Robustness and validity of all ML pipelines were confirmed through data augmentation, 10-fold cross-validation, batch normalisation and dropout techniques (see Supplementary Materials and Methods, ‘Machine learning’). The development and architecture of our model were crafted using PyTorch version 2.0.1, in the Python 3.9.0 environment. The final results were obtained by repeating the experiment five times to adjust hyperparameters.

### Statistical methods

The statistical approaches are summarised below. For full details of the model structures, *R*^2^ values, etc., see Supplementary Materials and Methods, ‘Statistics’.

#### Seismic signal differences between individual behaviours

Correlation of the dependent variables (amplitude, energy and frequency) was tested, and where correlation was strong (≥0.70), one variable was selected to represent both, to avoid redundant statistical testing. Here too, the PCA approach was considered but decided against because of the high values (≥0.7). Ultimately, we deemed that the simplicity of using the original variable outweighed the potential benefits of PCA in explaining additional variance. Only the energy and amplitude variables showed a correlation value ≥0.7. The energy variable (normalised energy) was consequently selected for further analysis because it was calculated using the full 0.4 s signal whereas the amplitude variable (peak amplitude) was derived from a singular peak. As such the energy variable represents the whole signal and was likely less sensitive to perturbations than the amplitude variable.

The differences in peak frequency and signal energy as a function of behaviour were tested with two linear mixed models (LMM_F_, LMM_LE_) that controlled for non-independence of signals from the same burrow. The assumption of normality was tested for both models, and where non-normality was observed, alternative dependent-variable transformations were attempted based on Box–Cox radar plots. The final energy model suffered from minor underdispersion, yielding conservative results. Within-individual signal parameter consistency was not explicitly tested.

*R*^2^ values indicated that both model structures were suitable, but behaviour explained a substantial amount of variance only in the energy model. Frequency was hence deemed uninformative for the purpose of distinguishing behaviours from signal features, and only the energy model (LMM_LE_) was considered further. A Tukey test was applied to the energy model for pairwise comparisons of the nine behaviours. Specifically, the Tukey–Kramer test was applied to adjust for unequal sample sizes.

#### Signal differences between behavioural groups

The energy differences between the three behavioural groups – non-percussive, body dropping and drumming – were assessed with one model (LMMG_LE_) that also controlled for non-independence of signals from the same burrow. The assumption of the normality was tested and alternative variable transformations were attempted based on a Box–Cox radar plot. The final behaviour group energy model suffered from minor underdispersion, yielding conservative results. A Tukey–Kramer test was applied this model to obtain pairwise behaviour group comparisons.

#### Signaller morphology and seismic signal features

The effect of crab morphology (claw size or carapace size) on the energy and peak frequency of seismic signals associated with the behavioural groups was tested with four interaction models (LMM_LEClaw_, LMM_LECar_, LMM_FClaw_, LMM_FCar_). In these models, the morphological features interacted with behavioural group and non-independence of multiple recordings from the same crab were controlled for. False discovery rates were controlled for using the Benjamini–Hochberg procedure for this four-model family (Benjamini and Hochberg, 1995). For all models, the normality of residuals was assessed, and variable transformations for the energy models and were informed by the Box–Cox radar plots. The final frequency models met the normality assumption, whereas the energy models suffered from minor underdispersion and thus yielded conservative results.

For both energy models (LMM_LEClaw_, LMM_LECar_), the interaction term was highly significant (*P*<0.001; Table S2) and six individual *post hoc* models (LMMD_EnergyClaw_, LMMD_EnergyCar_, LMMBD_EnergyClaw_, LMMBD_EnergyCar_, LMMNP_EnergyClaw_, LMMNP_EnergyCar_) further tested the effect of claw and carapace size on the energy of the three behavioural groups whilst controlling for repeated measures. As behaviour and morphology explained very little of the observed peak frequency variance, the effect of morphology on peak frequency was not explored further. Box–Cox radar plots were used to inform suitable variable transformations for the six *post hoc* energy models, normality of residuals was confirmed for all models, and the Benjamini–Hochberg procedure (Benjamini and Hochberg, 1995) was again applied to control false discovery rates in this family of six tests.

#### Signaller morphology and drumming spikes

Only the effect of claw size on drumming bout spikes was explored further because claw size was the only morphological feature with a significant effect on seismic drumming energy (see Table S2b). To assess the effect of claw size on the absolute amplitude of drumming spikes, drumming rate and bout size (=no. spikes), the dataset was firstly filtered to retain examples that were correctly assigned to the ‘drumming group’ by the ML algorithm (*n*=221) and had associated morphological crab measurements. In total, 161 drumming bouts were correctly identified and associated with 24 measured males. By only retaining correctly classified instances of drumming behaviours, we ensured that the analysed recordings had the characteristic spikes in the timeseries data. However, although the presence of these peaks was a requirement to meaningfully calculated the absolute amplitude of spikes and Δ*T* between spikes (outlined below), this step may have skewed the data to only retain the loudest drumming occurrences.

Drumming spikes were identified as absolute amplitude spikes in the close high-frequency geophone recording that exceeded 1/4 of the maximum absolute amplitude reading in the given bout and exceeded the absolute amplitude of the preceding and following 100 readings (i.e. 0.062 s). The effect of claw size on absolute spike amplitude was explored using all identified drumming spikes (*n*=1397). Drumming rate (=Δ*T* between absolute spikes in a singular bout) was calculated for all spikes except the first spike in a bout. Where multiple sequential bouts were accidentally captured as a singular drumming event, uncharacteristically large peak intervals were observed in recordings (Δ*T*>0.3 s). In case of this BORIS tagging error, the bout with the most spikes was retained for each tagged event, yielding *n*=1350 Δ*T* values.

The effect of claw size on absolute drumming spike amplitude and drumming rate was tested using two models (LMM_AmpDrum_, LMM_ΔtDrum_) that controlled for non-independence of repeated measures from each individual. Both models met the normality assumption. The effect of claw size on bout size was tested with a Poisson generalised linear mixed model (GLMM_BoutsizeDrum_) to analyse the count-based dependent variable and controlled for non-independence of repeated measures from each individual. The false discovery rate was controlled for using the Benjamini–Hochberg procedure for this family of three tests.

#### Drumming peak attenuation

For drumming behaviour, we further explored how peak frequency and signal energy was affected by propagation, and whether claw size (Claw.1×Claw.2; Fig. S1B) affected this change. Two interaction models (LMM_LEDistance_, LMM_FDistance_) tested the effect of distance to geophone on peak frequency and signal energy recorded by the far geophone pair (distance: 20–100 cm; Fig. 1). In these models, claw size and distance from the far geophone pair to the burrow interacted while we controlled for non-independence of measures from each individual. The assumption of normality was met for both models. The false discovery rate was controlled for using the Benjamini–Hochberg procedure for this family of two tests.

The fixed-effect variables in the frequency model explained <1% of the frequency variance, and propagation effects on frequency were not explored further. In the energy model, the interaction term was found to be non-significant, but the fixed effects explained a substantial amount of variance (17%). The interactive term was removed per the stepwise regression method, and claw size was removed because the independent effect of claw size on signal energy had already been tested in the previous section. The normality assumption was met for this final energy model (LMMLE_DistanceFinal_).

#### Environmental variables and seismic background noise

The effects of vegetation distance and wind speed on seismic background noise were analysed with an interaction model (LMM_NoiseInteraction_), a non-interaction model (LMM_Noise_) and a final wind-only model (LMM_WindNoise_), respectively. These three models all controlled for non-independence of measurements obtained from the same burrow and were attempted sequentially in this order per the stepwise regression method, as the interactive and vegetation distance terms proved non-significant in turn. The normality assumption was met for all three models.

#### Morphological variation on shoreline

To determine whether crab morphology affects their vertical position on the shoreline, claw size of males and carapace size of females were correlated with distance to vegetation (which occurred only on the upper shore) with two models (LMM_PositionMales_, LMM_PositionFemales_). Both models controlled for non-independence of crabs obtained from the same transect, and the normality assumption of both models was met. The false discovery rate was controlled for using the Benjamini–Hochberg procedure (Benjamini and Hochberg, 1995) of this family of two tests.

## RESULTS

### Crab courtship behaviour

Altogether, 8207 courtship behaviours and associated seismic recordings were analysed (excluding erroneous seismic recordings). Most common were non-percussive behaviours, followed by body dropping behaviours and drumming behaviours (Table S1).

Two behaviours involved impacts between body parts and the ground, or between two body parts. First, during aboveground drumming, the tip of the major claw was struck against the carapace. Second, during both body dropping behaviours (sequential and simultaneous), the carapace impacted the ground when it was rapidly lowered. Additional contact between the claw and ground was common for simultaneous waves and body drops, but was rare and appeared incidental for sequential waves and body drops. Although underground drumming likely also involved some impact given the associated seismic signal (outlined below), whether aboveground and underground drumming involved the same motions remains uncertain because the latter was not visually observed.

We observed a repetitive four-step courtship routine employed by males when females approached their burrow (Movie 1). First, males performed their low-intensity waving display. Second, males performed the sequential wave and body dropping motions (seqW+seqBD). Although these behaviours occur together as a claw wave and subsequent body drop, the individual components are henceforth referred to as ‘sequential waves’ and ‘sequential body drops’. Third, the simultaneous waves and body drops (simWBDs) were performed if the female approached further. Fourth, if females were near the burrow (generally <30 cm), males entered the burrow and started underground drumming (UGD).

Aboveground drumming (AD) was never part of these routines, but instead occurred as stand-alone events. Notably, during the 111 video recorded instances of aboveground drumming; the male's claw does not strike the ground. Instead, the tip of the major claw strikes the front of the carapace (Movie 2). Although aboveground drumming was approximately half as common as underground drumming (*n*_AD_=111, *n*_UGD_=219), their mean drumming rates were approximately equal at 0.16 and 0.15 s, respectively.

Introducing tethered females drastically increased the occurrence of simultaneous waves and body drops (*n*_PreTether_=32 versus *n*_Tether_=107) and underground drumming (*n*_PreTether_=19, *n*_Tether_=132), despite a lower mean duration of the tethered period compared with the pre-tether period (tether=928 s versus pre-tether=1179 s). See Movies 3 and 4 for videos of mud balling, locomotion and conflict.

### Seismic signal features of individual behaviours

**Fig. 2.**
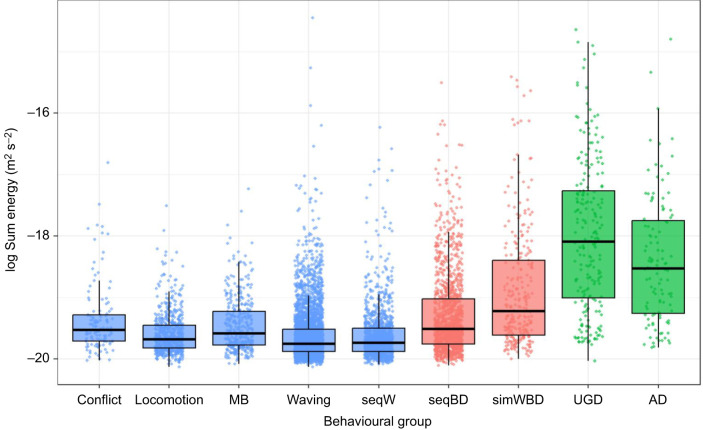
**Mean seismic energy levels associated with nine individual recorded behaviours.** Boxplots of the log sum energy of individual behaviours. For sample sizes, see Table S1. MB, mud balling; UGD, underground drumming; AD, aboveground drumming; simWBD, simultaneous wave and body drop; seqW, waving component of sequential wave and body drop; seqBD, body drop component of sequential wave and body drop. MB–conflict, seqBD–conflict, waving–locomotion, seqW–locomotion and seqW–waving were not significantly different (*P*>0.05). All other pairs were significantly different (*P*<0.05).

Signal loudness (referring to absolute peak amplitude and summed normalised energy) differed significantly between many behaviours when accounting for distance to the burrow, but peak frequency did not. Seismic energy of all non-percussive behaviours (waving, locomotion, conflict, mudballing, and the waving component of sequential waves and body drops) was generally low, with intermediate levels for body dropping behaviours (including simultaneous wave and body drop, and the body dropping component of sequential waves and body drops), and the highest energy levels for drumming behaviours (aboveground and underground drumming; Fig. 2). The Tukey–Kramer test for LMM_LE_ further demonstrates that seismic energy differed significantly amongst the behavioural groups. All individual behaviours constituting the drumming and body dropping groups were significantly different (*P<*0.05; Fig. 2; Fig. S2). Energy levels recorded for individual body dropping and drumming behaviours differed significantly from all individual non-percussive behaviours (where *P<*0.05), except sequential body drops and conflict (*P*=0.93; Fig. 2; Fig. S2). Non-significant energy differences were common among non-percussive behaviours (*P*>0.05 for mud balling and conflict, waving and locomotion, sequential wave and locomotion, sequential wave and waving; Fig. 2; Fig. S2). As frequency variance was poorly described by the behaviour fixed-effect variable (LMM_F_: marginal *R*^2^<0.02; Supplementary Materials and Methods), we concluded that peak frequency was not meaningfully different between behaviours.

**Fig. 3.**
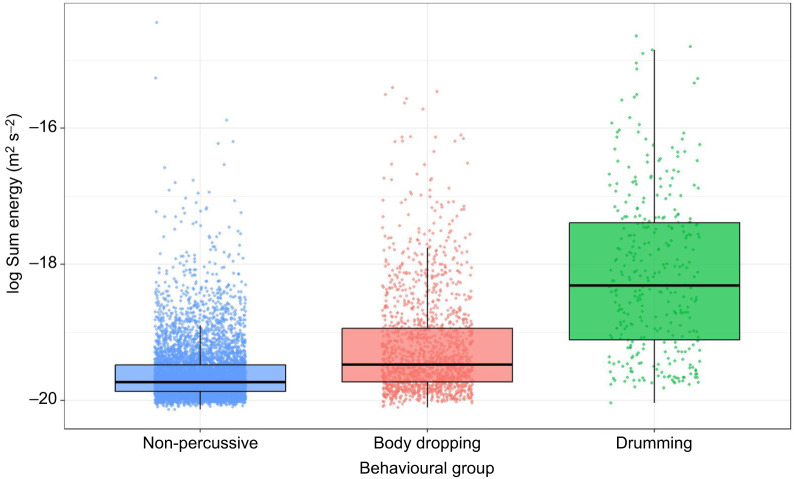
**Mean seismic energy levels associated with three behaviour groups.** Boxplot of log sum energy of behavioural groups. Non-percussive group: conflict, locomotion, MB, waving and seqW (*n*=6359). Body dropping group: simWBD and seqBD (*n*=1518). Drumming group: AD, UGD behaviours (*n*=330). *P*<0.05 for all group comparisons per Tukey–Kramer outputs.

The seismic energy of signals escalates through the four-step courtship routine (Fig. 2). The standard, low-intensity waving displays (waving and sequential waves) produced minimal seismic energy, followed by a substantial increase of seismic energy associated with the sequential body dropping, and another increase of seismic energy for simultaneous waves and body drops. The greatest amount of seismic energy was caused by underground drumming. However, drumming often captured two spikes (i.e. two drums) in the 0.4 s standardised window compared with a singular pulse for body drops, which likely increased energy levels for drumming compared with body drops (Figs 2 and 3).

### Seismic signal features of grouped behaviours

When behaviours were grouped, seismic energy levels were statistically different between all behavioural groups (*P*<0.05). The drumming group produced the highest seismic energy levels, followed by the body dropping group and the non-percussive group, based on LMMG_LE_ (Fig. 3). The widths of confidence intervals of the associated Tukey–Kramer test on the grouped behaviours were much smaller and further separated than when behaviours were explored individually (Figs S2 and S3). The energy levels of all three behavioural groups were thus highly significantly different (Fig. 3; Fig. S3).

### Behavioural classification using ML on seismic spectrograms

**Fig. 4.**
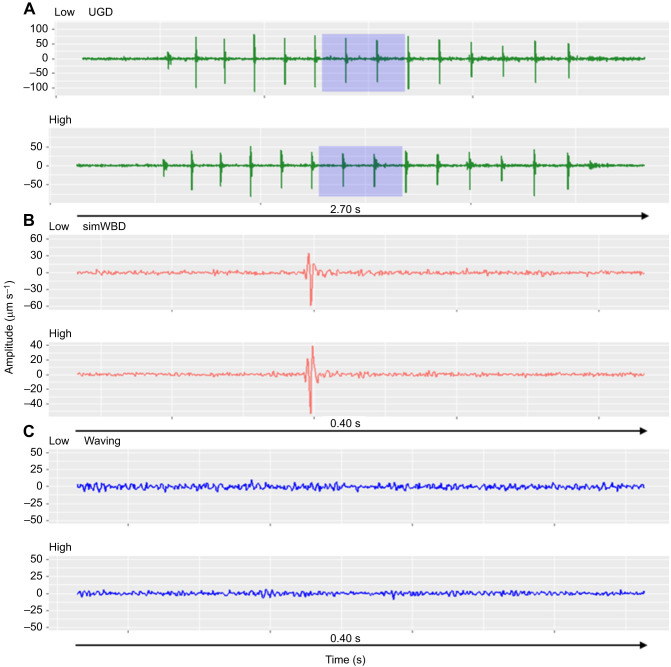
**Exemplar seismic recordings (velocity) of drumming (e.g. UGD), body dropping (e.g. simWBD) and non-percussive behaviours (e.g. waving) correctly identified by machine learning (ML) model.** (A) Recording of underground drumming behaviour. Blue shading indicates 0.4 s section used to make spectrograms, peak amplitude and calculate variables outlined in the Materials and Methods. (B) Recording of body dropping behaviour per point tag ±0.2 s. (C) Recording of body dropping behaviour per point tag ±0.2 s. Spectrograms of these examples are shown in Fig. S4.

Automated classification using our ML algorithms and two sets of spectrograms yielded the highest correct classification rates using the WL=128 combined spectrograms for individual behaviours (51.97%), and the WL=256 high-frequency spectrograms for the behavioural groups (70.03%, Table 1). Assuming nine bins (one per behaviour), random designation of a spectrogram to one of nine bins should correctly classify ∼11.11%. Likewise, assuming three bins (one per behavioural group), random designation of spectrograms to one of three bins should correctly classify ∼33.33%. Instead, when using ML, we observed means of 34.22% and 70.03% correct classification for the WL=256 spectrograms, and means of 36.6% and 60.0% for the WL=128 spectrograms. Mean accuracy was obtained by taking the mean percentage of images correctly designated to their behaviour or group by the ResNet models across the 10 folds of the cross-validation process. Based on odds ratios, the ResNet model had approximately four times better odds of classifying a behaviour correctly than chance when using the spectrogram parameter WL=256. With spectrogram parameter WL=128, odds ratios were much more variable (ranging from 2.9 to 8.7). The greatest improvement relative to chance was observed for the individual behaviour model using the combined spectrograms with WL=128 (odds ratio=8.7), but the highest absolute rate of correct classification was observed for the behavioural group model using the high frequency spectrogram only (70.04% correct classification). See Fig. 4 for exemplar waveforms of the non-percussive, drumming and body dropping groups.

**Table 1.** Automated behavioural classification results

		Chance						ResNet model					
		Individual behaviours			Behavioural groups			Individual behaviours			Behavioural groups		
Geophone type		Low	High	Comb	Low	High	Comb	Low	High	Comb	Low	High	Comb
WL=256	Mean accuracy (%)	11.11 (calculated)			33.33 (calculated)			31.76	34.22	33.18	66.54	70.03	68.68
	Odds ratio	N/A			N/A			3.7	4.2	4.0	4.0	4.7	4.4
WL=128	Mean accuracy (%)	11.11 (calculated)			33.33 (calculated)			26.87	31.08	51.97	52.96	63.30	63.81
	Odds ratio	N/A			N/A			2.9	3.6	8.7	2.3	3.4	3.5
	Sample size range	888–1318			2640–3036			888–1318			2640–3036		

WL, window length in samples. Low, 4.5–160 Hz spectrogram; High, 100–400 Hz spectrogram; Comb, high- and low-frequency spectrograms combined. Behavioural groups: drumming, body dropping, non-percussive (see Fig. 3). For the ResNet columns, mean accuracy is obtained by calculating the mean percentage of images correctly classified across all 10-folds of the cross-validation process. Odds ratios of correct classification are calculated using the mean accuracy % for the ResNet models and the corresponding chance values. Sample size range indicated for all groups after sample size adjustments noted in the Materials and Methods.

The seismic recordings of drumming behaviours correctly identified by the algorithm have repeating high amplitude spikes (i.e. temporally short pulse) in the time series data (Fig. 4A), with the highest amplitudes recorded by the high-frequency geophones (median absolute amplitude of close low-frequency geophone=3.6e–5 m s^−1^ and IQR=4.0e–5 m s^−1^; median absolute amplitude of close high-frequency geophone=4.35e–5 m s^−1^ and IQR=3.8e–5 m s^−1^). Consecutive drumming peaks occurred at short intervals (median=0.16 s, IQR=0.03 s) for a median eight beats (IQR=5). Absolute amplitude of spikes in the middle of bouts varied based on visual inspection of the time series data, but the first and last spikes were generally lowest in absolute amplitudes.

The seismic recordings of body dropping behaviours were often marked by a singular sharp spike in the 0.4 s recording (Fig. 4A,B). The absolute amplitude of body dropping spikes was lower than drumming spikes, and the highest absolute amplitude values were recorded by the low-frequency geophones (median absolute amplitude close low frequency geophone=1.05e–5 m s^−1^ and IQR=6.48e–6 m s^−1^; median absolute amplitude of close high frequency geophone=7.20e–6 m s^−1^ and IQR=7.18e–6 m s^−1^). In spectrograms, body dropping often appeared as singular broadband spikes, but the broadband signals were generally less clear for sequential body drops than simultaneous waves and body drops.

The seismic recordings of non-percussive behaviours showed negligible seismic activity without pulses or spikes (Fig. 4).

### Effect of signaller morphology on seismic signal features

The effect of claw and carapace size on signal energy differed between behavioural groups (LMM_LEClaw_, LMM_LECar_), but the effect of claw and carapace size on peak frequency did not differ between behavioural groups (LMM_FClaw_, LMM_FCar_). Claw and carapace size significantly interacted with behaviour group in the energy models (LMM_LEClaw_: marginal *R*^2^=0.26, conditional *R*^2^=0.57, d.f.=4756, *t*=6.93, *P*<0.001, rank=1, BH critical value=0.025, and LMM_LECar_: marginal *R*^2^=0.24, conditional *R*^2^=0.59, d.f.=4380, *t*=4.63, *P*<0.001, rank=2, BH critical value=0.05), but claw and carapace size did not significantly interact with behaviour group in the frequency models (LMM_Fclaw_ and LMM_Fcar_; Table S2A).

**Fig. 5.**
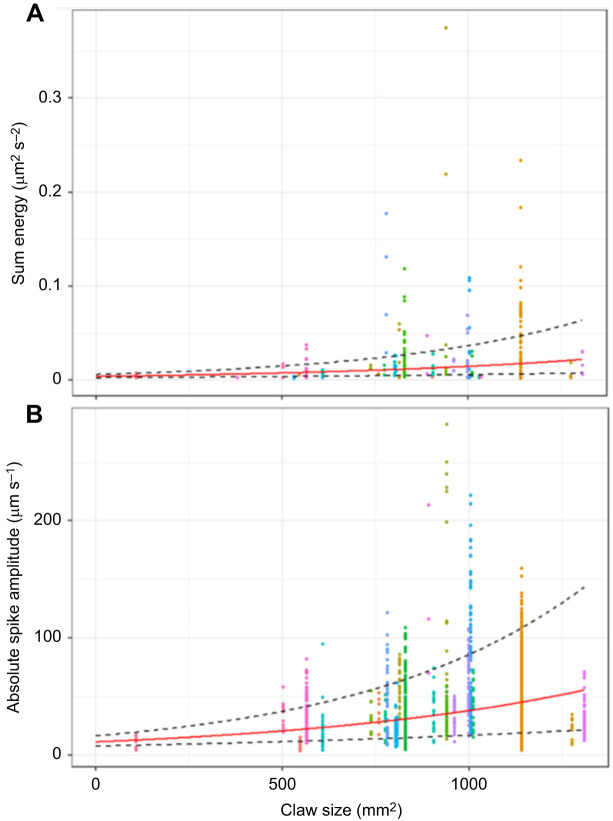
**Effect of claw size on normalised seismic drumming energy and absolute drumming spike amplitudes.** (A) Sum normalised energy of drumming as a function of claw size. *n*=227 drumming bouts; *n*=30 crabs, indicated by unique colours. Correlation estimate (red) and error lines (±s.e.) calculated by back transformation of LMM_EnergyClaw_ estimates; *P*=0.016. Measurements obtained from 0.4 s middle sections of recordings corresponding to the spectrograms used by the ML algorithm. (B) Drumming peak absolute amplitude (velocity) as a function of claw size. *n*=161 drumming bouts; *n*=24 crabs, indicated by unique colours. Correlation estimate (red) and error lines (±s.e.) calculated by back-transformation of LMM_AmpDrum_ estimates; *P*=0.011. Measurements from close high-frequency geophone recordings, of full drumming recordings.

Claw size was significantly positively correlated with sum normalised energy in the seismic drumming group signals (LMM_EnergyClaw_: marginal *R*^2^=0.08, conditional *R*^2^=0.26, *t*=2.63, d.f.=16.15, *P*=0.016, rank=1, BH critical value=0.05; Fig. 5A). However, claw size did not affect signal energy for body dropping and non-percussive behaviour groups, and carapace size did not affect the energy of any behaviour group (for remaining results, see Table S2B).

Based on Fig. 5A, claw size outliers were removed by retaining the middle 90% of claw size variance (individuals with claw size 108, 1277 and 1308 were removed) and LMM_EnergyClaw_ was re-run. Considered independently, this model indicates that effect of claw size on sum normalised energy remained significant (*P*=0.024) and the correlation remained positive.

### Effect of claw size on drumming spikes

Claw size significantly and positively affected log absolute amplitude of all drum spikes identified using the recording of the close high-frequency geophone (LMM_AmpDrum_: marginal *R*^2^=0.09, conditional *R*^2^=0.45, *t*=2.81, *P*=0.011, rank=1, BH critical value=0.05; Fig. 5B). However, claw size did not significantly affect bout size or drumming rate (GLMM_BoutsizeDrum_ and LMM_ΔtDrum_; Table S2C).

Based on Fig. 5B, the claw size outliers were removed by retaining the middle 90% of variance (individuals with claw size 108 and 1308 were removed) and LMM_AmpDrum_ was re-run. Considered independently, this model indicates that effect of claw size on absolute spike amplitude was marginally non-significant (*P*=0.08) but the direction of the effect remained positive.

### Effect of propagation distance on seismic signal features

**Fig. 6.**
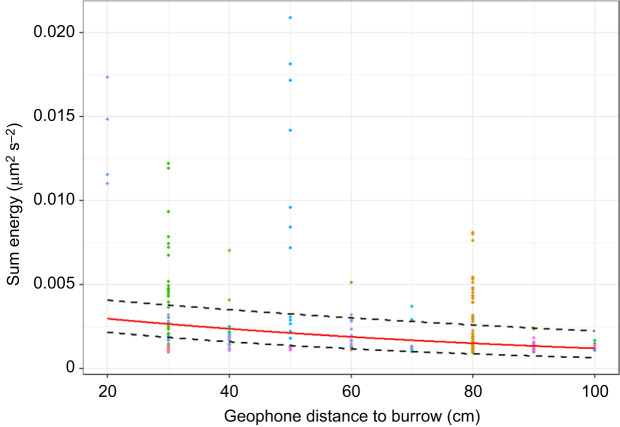
**Seismic normalised energy of drumming signal as a function of sensor distance to burrow entrance.** *n*=30 focal burrows, indicated by different colours; *n*=227 drumming bouts. Distance to burrow is a proxy for distance to signalling crab. Correlation (red) and error lines (±s.e.) calculated by back-transformation of LMMLE_DistanceFinal_ outputs; *P*=0.005. Measurements obtained from 0.4 s middle sections of recordings corresponding to the spectrograms used by the ML algorithm.

The seismic energy of drumming signals decreases significantly over distance (LMMLE_DistanceFinal_: marginal *R*^2^=0.13, conditional *R*^2^=0.49, *t*=−3.01, *P*=0.005; Fig. 6). Notably, this trend remains significant without the 20 cm data (*t*=−2.317, *P*=0.027). The lack of a significant interaction between distance and claw size shows that the energy decrease is unaffected by claw size (LMM_LEDistance_, marginal *R*^2^=0.17, conditional *R*^2^=0.50, *t*=−1.07, *P*=0.30, rank=1, BH critical value=0.05). Furthermore, because the interactive term between distance and claw size collectively described <1% of variance in the frequency model (LMM_FDistance_), distance to burrow and claw size do not meaningfully affect peak frequency. Note that only drumming behaviours were assessed in this context because the non-percussive behaviours did not produce seismic signatures, and crab morphology had no effect on body dropping signatures, thus not permitting an interactive statistical assessment.

Because the small sample at 20 cm appeared to drive much of the correlation, the model was re-run without it. Because the trend remained unchanged (*t*=−2.317, *P*=0.027), the full dataset was considered going forward.

### Effect of environmental variables on seismic noise and crab distribution

**Fig. 7.**
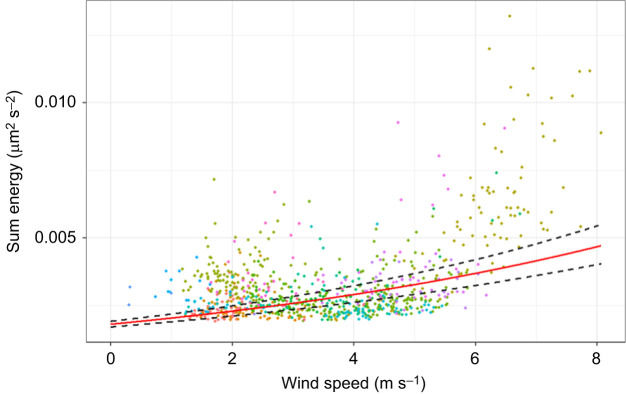
**Seismic energy as a function of wind speed.** *n*=27 focal burrows, indicated by different colours; *n*=831 wind recordings. Correlation estimate (red) and error lines (±s.e.) calculated by back-transformation of LMMLE_WindNoise_ outputs; *P<*0.001. Measurements obtained from 0.4 s sections of recordings corresponding to waving spectrograms correctly identified by the ML algorithm. Four outliers noted above are not included.

Seismic background noise levels (energy) were positively correlated with wind speed (LMM_WindNoise_: marginal *R*^2^=0.33, conditional *R*^2^=0.65, *t*=10.11, *P*<0.001; Fig. 7). The effect of wind on noise did not change significantly as a function of vegetation distance (LMM_NoiseInteraction_; Table S2D). When the effect of wind was also considered non-interactively, the effect of vegetation distance on noise levels remained non-significant (LMM_Noise_; Table S2D). Finally, neither the claw size of males nor the carapace size of females had a significant effect on the distance of the crab to the vegetation on the upper shore (LMM_PositionMales_, LMM_PositionFemales_, Table S2E).

## DISCUSSION

### *In situ* recording of seismic communications

Seismic animal communication remains understudied in the wild (Hill, 2008; Hill and Wessel, 2016). Inspired by studies of vocally generated seismic signals of elephants (Günther et al., 2004; Mortimer et al., 2018), we used geophones to record percussive seismic signalling between 4.5 and 400 Hz by wild *A. tangeri*. Over 8000 seismic and video behaviour recordings were obtained. This is the first time that geophones have been used to seismically record intertidal invertebrates *in situ*. Natural ground substrates are both harder to vibrate and more heterogeneous than air, which theoretically distorts seismic signals more severely than sounds during propagation (Arnason et al., 2002; Margerin, 2011; Snieder, 1986). Because natural substrates are (near-)impossible to replicate in the laboratory, our findings show that geophones are valuable for studies of other animals that also occupy sandy substrates. However, our spectrograms indicate that percussive signals exceeded 400 Hz and future percussive studies should instead use geophones ranging from 4.5 to 750 Hz (Mowles et al., 2017; Takeshita and Murai, 2016).

### Differentiating seismic behaviours using basic signal features

Two variations of percussive seismic signalling were observed: (1) direct percussion, where the ground is struck with the carapace (and claw); and (2) indirect percussion, where the carapace was struck by the claw, after which the vibrations were transferred into the ground. Direct percussion was associated with body dropping behaviours. In videos of both sequential and simultaneous waving and body dropping, the ground was percussed by the carapace when it was rapidly lowered. For simultaneous waving and body dropping, the claw also commonly impacted the ground. However, for the sequential behaviour, the ground was generally not impacted by the claw, and it appeared incidental when it did occur. Collectively, the body dropping behaviours resemble the abdomen drumming behaviours of other arthropods, including the male wolf spider *Hygrolycosa rubrofasciata*, which drums dry leaves with its abdomen (Hill, 2008; Rivero et al., 2000). Indirect seismic percussion was associated with drumming behaviours, but acoustic vibrations were also easily audible in the videos. This method of indirect seismic vibration production notably contradicts prior observations of *A. tangeri* striking the substrate with its claw during drumming (Salmon and Atsaides, 1968; Wolfrath, 1993). Instead, our findings resemble the indirect seismic drumming observed for other fiddler crab species (Mowles et al., 2017), albeit without apparent stridulation.

The courtship signals of *A. tangeri* males followed a systematic four-step routine: (1) waving, (2) sequential waves and body drops, (3) simultaneous waves and body drops, and (4) underground drumming (Movie 1; Table 1). The distinction between steps 2 and 3 has not been described previously for this species and were instead collectively called ‘high intensity waving’ (Crane, 1975; Jordão et al., 2007; Salmon and Atsaides, 1969). Aboveground drumming was not part of these routines and instead occurred as a stand-alone behaviour, unlike for other fiddler crab species (Mowles et al., 2017).

The routine was marked by a seismic escalation where negligible seismic energy was associated with: (1) waving, followed by a notable seismic signal for sequential body drops (2), a further energetic increase for (3) simultaneous waves and body drops, and the loudest signals associated with (4) underground drumming (Fig. 1; Fig. S2). The differences were exacerbated when behaviours were designated into non-percussive, body dropping and drumming behavioural groups (Fig. 3; Fig. S3). These routines as a whole cover the visual, acoustic and seismic modalities, and part of these routines thus remains detectable even if two senses suffer from noise. In addition, the routine is marked by the introduction of vibrational signals as mating potential increases. Here step 1 is visual, steps 2 and 3 are visual and vibrational, and step 4 is vibrational. This shift towards percussive signals suggests that, as in other invertebrates (Rundus et al., 2011), the role of seismic signals may be important for sexual competition. Alternatively, given the energetic costs of percussive vibrational signalling in small-bodied animals (Davranoglou et al., 2019), seismic signals may be reserved for when females are close. Conserved energy can be allocated to waving signal quality (Takeshita and Murai, 2016), or growth and potentially louder signals (Fig. 5B) in the long term.

The ability to control the energy dedicated to seismic signals is perhaps best demonstrated by the energetic differences between sequential and simultaneous waves and body drops (Fig. 2). Claw waves are only converted into seismic energy when concurrent with a body drop. This is likely because waves result in claw–ground contact in simultaneous, but not sequential, waving and body dropping. The utility of the seismic component of these waves is likely two-fold. First, the visual and seismic components may compound as complementary signals to attract passing females and deter potential competitors that also see the waving display (Krebs and Davies, 1978; Mitoyen et al., 2019). Second, the information conveyed via the visual and seismic waving component of simultaneous waving and body dropping may be redundant (Krebs and Davies, 1978; Mowles et al., 2017). This ensures that individuals facing away, or obscured by vegetation, are also targeted. In theory, this reduces the futile incurrence of energetic costs (Krebs and Davies, 1978).

### Automated behavioural classification

Machine learning shows promise for automated behavioural classification of percussive fiddler crab behaviours based on seismic spectrograms, but requires improvements before future studies can forego manual behaviour coding. Automated classification using the ResNet model was most successful in absolute terms (70% correct classification rate) when applied exclusively to close high-frequency WL=256 spectrograms of grouped behaviours, and relative to the chance base line in case of the combined WL=128 spectrograms of individual behaviours (odds ratio=8.7).

Based on inspection of the correctly classified behavioural group WL=256 spectrograms (Fig. S4), this improvement was possibly because drumming and body dropping signals commonly spanned the full frequency range of high-frequency spectrograms (100–400 Hz). Consequently, the temporal discrepancies were particularly noticeable, with spectrograms commonly capturing multiple drums but not multiple body drops. Nonetheless, compared with the WL=128 spectrograms with coarser frequency resolution, correct classification improved by ∼7% with WL=256 spectrograms. As such, frequency may play some role in discriminating these behaviours, but this could not be explored in greater detail owing to the 0.4 s signal durations, which limited spectrogram window lengths. The improvement of correct classification rates of grouped behaviours compared with individual behaviours reiterates that many individual behaviours were not meaningfully different in the seismic domain and mirrors the energetic modelling results (Figs 2 and 3).

Temporal characteristics such as rhythm and potentially spike width (most clearly captured by high-frequency spectrograms) additionally appeared more important in classifying behavioural groups than frequency content, because high-frequency WL=256 spectrograms alone worked better for automated behaviour classification than high- and low-frequency WL=256 spectrograms combined (Table 1). If fundamental frequency differed substantially between drumming and body dropping behaviour groups, this would have been reflected by the low-frequency geophone and should have improved the classification outcomes.

Interestingly, classification of individual behaviours improved drastically when the high- and low-frequency spectrograms were both considered for spectrograms with WL=128 compared with WL=256. This primarily suggests that the temporal difference between individual behaviours was exacerbated when the temporal resolution of the signal was increased. Notably, however, this did not occur when the low- and high-frequency components of signals were considered. Further studies using seismic sensors that have a wider sensitivity range to capture the full signals may provide insights as to why this occurred.

The ML approach appeared to be limited. The remaining incorrectly classified spectrograms (≥29.07%), and variability amongst correctly classified spectrograms of behaviour groups, demonstrates the challenge when classifying seismic crab signals. Other issues include the close proximity of other individuals, which likely created interfering signals, while passing people and other animals generated interfering footfall signatures (Szenicer et al., 2021). Altogether, these findings indicate that ML approaches may be used to coarsely classify several fiddler crab behaviours using seismic recordings alone, but noise and overlapping signal features caused non-insignificant rates of incorrect classification. Automated seismic species classification may likewise prove difficult for co-occurring species with overlapping vibrational signalling niches, such as the wolf spiders *Schizocosa stridulans* and *Schizocosa uetzi* (Choi et al., 2024).

Ultimately, we tentatively accept alternative hypothesis 1, that the basic features of percussive seismic recordings vary with behaviour, and percussive behaviours can be classified using a ML model based on seismic spectrograms. Seismic energy and peak amplitude differed significantly between behaviours, and percussive seismic behaviour groups were roughly classifiable using a ML model. Nonetheless, although this approach may prove useful in automated signal detection in future, it requires further fine-tuning to reduce incorrect classification rates from 30%. Ultimately, *A. tangeri* seismically convey distinct information through controlling the rhythmic content and loudness of percussive signals, much like as observed for kangaroo rats (Randall, 1997; Randall and Matocq, 1997).

### Competitive seismic advantage of larger claws

Our results indicate that although larger claws may carry various seismic signalling advantages, these advantages are limited to drumming signals amongst the tested behaviours. Specifically, when considering all individuals, larger claws produce higher energy drumming signals and higher amplitude drumming spikes (LMM_LEClaw_, LMM_EnergyClaw_; Table S2A,B). Louder drumming signals from larger claws may be expected owing to the functional role of the claw as the ‘hammer’ in drumming signal production. However, more data for crabs with extreme claw sizes are required to draw definitive conclusions owing to the effect of outliers in our study. Specifically, significance of the correlation between claw size and drumming spike amplitude and normalised signal energy was reduced when extremely large- and small-clawed individuals were excluded. Additionally, despite the functional role of the carapace as the ‘anvil’ in aboveground drumming, signal loudness and peak frequency of all behaviour groups were surprisingly unaffected by carapace size (LMMF_Claw_ and LMMF_Car_; Table S2A).

The theoretical advantages of louder percussive signals are numerous. First, louder signals can better overcome seismic wind noise, which is generally comparable in energy to drumming signals at distances ≥20 cm (Figs 6 and 7). Second, louder seismic signals could overpower signals (visual and/or vibrational) produced by neighbouring competing males to retain female interest, even if the male that is in the burrow cannot do this through visual means. Third, larger crabs may utilise louder drumming to reach more distant females. Finally, larger claws are associated with increased female interest (Callander et al., 2012; Oliveira and Custódio, 1998), and aggressive visual displays with larger claws can avert physical conflict with competing males (Bywater et al., 2018). If additional outlier data confirm our observations, then louder drumming signals may also serve as stronger deterrents to males and attractants to females. Seismic and acoustic playback studies could uncover female preferences associated with percussive signal features in the absence of their visual counterparts. Playbacks could likewise test whether large-clawed individuals enjoy percussive communication advantages in variable noise levels.

Claw size was expected to positively affect the bout length and drumming rate as observed in *Astruca lactea*, where higher drumming rates were associated with male fitness and female preference (Takeshita and Murai, 2016). However, this relationship between claw size and drumming signals was not observed for *A. tangeri* (GLMM_BoutsizeDrum_ and LMM_ΔtDrum_; Table S2C). This may be due to the stridulatory production method of vibrational drumming by *A. lactea* (Takeshita and Murai, 2016), compared with the percussive production method for *A. tangeri*.

Ultimately, signal loudness/amplitude was affected by signaller morphology and we therefore accepted hypothesis 2, that percussive seismic signal features are affected by signaller morphology. This suggests that seismic signal loudness may serve as an honest signal for females to assess male quality and claw size without the need for visual confirmation (Krebs and Davies, 1978).

### Environmental effects on seismic signals

Faster winds increased seismic noise in the environment (LMM_WindNoise_; Fig. 7), but noise was unrelated to proximity to vegetation (LMM_NoiseInteraction_, LMM_Noise_; Table S2D). This was surprising given that grass hummocks in the desert are associated with higher noise levels, and act as substrate excitors when vibrated by the wind (Mason and Narins, 2002). A potential cause of this is the difference in substrate moisture content and density of sand particles between desert environments and the intertidal zone. In this study, sand was densely packed and bound by water, leading to an increase in the energetic input required to induce a detectable vibration (Aicher and Tautz, 1990). The fact that sections of the shoreline do not carry a competitive advantage for seismic signalling is mirrored by the random distribution of crab sizes as a function of proximity to vegetation (Table S2E). However, we cannot eliminate the possibility that geophones were themselves vibrated by the wind. Retesting using geophones that can be buried in full should provide optimal insights.

Seismic drumming signals attenuate over distance irrespective of claw size (LMMLE_DistanceFinal_; Fig. 5, 10 cm, Fig. 6, 20–100 cm), and peak frequency is not altered over distance (LMMF_Distance_). Ultimately, the environment drives attenuation, but peak frequency was not meaningfully altered by the environment or controlled by the signaller. A sandy substrate thus appeared to impose distance constraints on seismic communication, which is likewise observed in seismic communication studies of megafauna (Arnason et al., 2002; Mortimer et al., 2018).

In short, we partly accept hypothesis 3, that seismic signals are influenced by abiotic factors. Seismic signals do change over distance, and seismic noise is affected by wind speed, but proximity to vegetation is not associated with wind noise levels, and claw size does not affect signal attenuation or peak frequency of signals over distance.

### Constraints on percussive seismic signals

The frequency domain of percussive seismic signals contained little information about morphology or behaviour in *A. tangeri*. Peak frequency of seismic signals was very poorly described by individual behaviours, and the ML outputs suggest that the percussive seismic signals were all broadband without frequency modulation through time. Although high-frequency geophones exceeding 400 Hz may have the detected differences in upper frequency ranges, our data suggest that the lack of a behavioural effect on frequency content is likely due to biomechanical constraints on percussion, and the impulsive nature of the vibration generation. Further, the frequency content of signals is commonly affected by the percussed structure, which may be significant owing to resonance. For *A. tangeri*, however, we did not observe frequency filtering. The broadband spikes were instead comparable to those observed for other small animals that communicate using percussive signals, such as the kangaroo rat (Randall, 1997) and invertebrates on non-ground substrates (e.g. plant hoppers; Davranoglou et al., 2019; Žunič et al., 2008). Broadband signals thus recur in percussive seismic communication across species regardless of substrate, animal morphology, behavioural specifics, and aboveground or belowground production. Surprisingly, our results suggest that percussive *A. tangeri* signals remain broadband even when the vibrations are induced indirectly (e.g. when the signaller's own carapace is impacted). However, in the contexts of multimodal and unimodal signalling, frequency variations may not be required. In combination with visual displays (i.e. waving and body dropping), a broadband signal could maximise female attention, whereas a seismic signal on its own (i.e. underground drumming) could be used to assess males that are visually obscured. Crucially, aboveground drumming is also less visually elaborate than the waving and body dropping behaviours, and reportedly replaces waving displays at night (Wolfrath, 1993). Drumming may hence be preferentially performed when visual communication is compromised. Despite our finding, broadband percussion is not universal. In jumping spiders (*Habronattus dossenus*), formants are observed where the substrate (stretched nylon fabric) is percussed with the front legs (Elias et al., 2003). However, given that the percussive signals of another spider (*Schizocosa retorsa*) are broadband on some substrates, this suggests that there may be a continuum of percussive complexity, which may be substrate driven (Elias et al., 2003; Hebets et al., 2008).

Small animals are constrained in the vibration generation mechanisms they can employ using their muscles owing to the relationship between muscle size and force, and percussion may hence be particularly advantageous in this context (Ilton et al., 2018). Some signalling methods are only attainable for larger animals, e.g. elephants, lions and rhinos vocally produce frequency-modulated seismic signals (O'Connell-Rodwell et al., 2001; Reinwald et al., 2021; Szenicer et al., 2021). Loud vibrational signalling by small animals instead often relies on specialised vibration generation organs that make use of power amplification mechanisms, such as springs, to overcome size constraints (Davranoglou et al., 2019; Patek et al., 2011). Percussion may be a simple yet effective signalling method for small animals but can restrict the information they can encode through frequency content. Although we did not observe an effect of morphology on frequency content, animals could theoretically shape frequency content of their percussive signals via indirect percussion, where resonance of body parts could influence the frequency content transmitted into the substrate.

Crucially, broadband percussive signals are advantageous in some situations as they are more robust for information transfer in the face of variable environmental noise and frequency filtering by the substrate compared with narrow-band signals (Mortimer, 2017). Therefore, broadband signals are more likely to reach conspecifics when a small animal aims to communicate seismically. In addition, constraints acting on the biomechanics of percussive signals may induce honest signalling. Signal loudness is theoretically constrained according to the ‘hammer's mass’ (e.g. claw size) and the animal's physical condition, meaning it could serve as an honest signal for mate-searching females (Krebs and Davies, 1978).

### Conclusions

Communication via substrate vibrations is commonplace amongst animals, but large seismic communication datasets of wild invertebrates remain rare. The present study demonstrates that geophones are effective for seismically recording wild invertebrates, and that these recordings could be used to classify courtship behaviours, including by use of an automated ML algorithm.

For male *A. tangeri*, we established that percussive seismic signals are only produced by body dropping and drumming behaviours, which are generated directly by impacting the ground with the carapace, and indirectly by impacting the signaller's carapace with the claw. Seismic energy escalation is observed through the described courtship routine, which may maximise female attention in combination with visual signals. The loudness of drumming signals appeared to be affected by male claw size (albeit less so when individuals with extreme claw sizes were excluded), whereas peak frequency was unaffected by morphology and propagation, and was unmodulated between behaviours. This may be another potential advantage for larger-clawed males, as louder signals produce higher signal-to-noise ratios and can overcome the effects of background noise. Indeed, the natural environment of fiddler crabs proved seismically noisy owing to wind, but wind noise did not increase as a result of vegetation proximity.

Inherent to their biomechanics, percussive seismic signals appeared limited in complexity. Our work on male fiddler crabs indicates some constraints acting on percussive signalling. Most prominently, we found that percussive seismic signallers appear to have no control over frequency content through modulating behaviour or through differences in body morphology, yielding broadband seismic signals. This limits information content about the signaller in the frequency domain when compared with seismic signals produced via vocalisations. Percussive seismic signalling instead appears to rely on rhythmic and loudness manipulations. However, we cannot explicitly rule out that this may be an artifact of our spectrogram parameters, which were limited in frequency resolution because of short signal lengths. Crucially, the broadband percussive signals also have advantages as they facilitate robust information transfer across a variety of physical environments and noise conditions. For small animals such as *A. tangeri*, percussive signals thus provide a simple, but effective way to communicate in a noisy environment despite constraints imposed by their size.

## Supplementary Material

10.1242/jexbio.249323_sup1 Supplementary information

## References

[JEB249323C1] Aicher, B. and Tautz, J. (1990). Vibrational communication in the fiddler crab, *Uca pugilator*. *J. Comp. Physiol. A* 166, 345-353. 10.1007/bf00204807

[JEB249323C2] Arnason, B. T., O'Connell-Rodwell, C. E. and Hart, L. A. (2002). The properties of geophysical fields and their effects on elephants and other animals. *J. Comp. Psychol.* 116, 123-132. 10.1037/0735-7036.116.2.123 12083604

[JEB249323C3] Barnett, K. E., Cocroft, R. B. and Fleishman, L. J. (1999). Possible communication by substrate vibration in a chameleon. *Copeia* 1999, 225-228. 10.2307/1447408

[JEB249323C4] Benjamini, Y. and Hochberg, Y. (1995). Controlling the false discovery rate: a practical and powerful approach to multiple testing. *J. R. Stat. Soc. B* 57, 289-300.

[JEB249323C5] Bywater, C. L., Wilson, R. S., Monro, K. and White, C. R. (2018). Legs of male fiddler crabs evolved to compensate for claw exaggeration and enhance claw functionality during waving displays. *Evolution (N. Y)* 72, 2491-2502. 10.1111/evo.13617 30284733

[JEB249323C6] Callander, S., Jennions, M. D. and Backwell, P. R. Y. (2012). The effect of claw size and wave rate on female choice in a fiddler crab. *J. Ethol.* 30, 151-155. 10.1007/s10164-011-0309-6

[JEB249323C7] Choi, N., Miller, P. and Hebets, E. A. (2024). Vibroscape analysis reveals acoustic niche overlap and plastic alteration of vibratory courtship signals in ground-dwelling wolf spiders. *Commun. Biol.* 7, 1-13. 10.1038/s42003-023-05700-6 38182735 PMC10770364

[JEB249323C8] Crane, J. (1975). *Fiddler Crabs of the World*. Princeton University Press.

[JEB249323C9] Darden, S. K., May, M. K., Boyland, N. K. and Dabelsteen, T. (2019). Territorial defense in a network: audiences only matter to male fiddler crabs primed for confrontation. *Behav. Ecol.* 30, 336-340. 10.1093/beheco/ary169

[JEB249323C10] Davranoglou, L. R., Cicirello, A., Taylor, G. K. and Mortimer, B. (2019). Planthopper bugs use a fast, cyclic elastic recoil mechanism for effective vibrational communication at small body size. *PLoS Biol.* 17, 1-17. 10.1371/journal.pbio.3000155 PMC6413918 30860993

[JEB249323C11] Elias, D. O., Mason, A. C., Maddison, W. P. and Hoy, R. R. (2003). Seismic signals in a courting male jumping spider (Araneae: Salticidae). *J. Exp. Biol.* 206, 4029-4039. 10.1242/jeb.00634 14555743

[JEB249323C12] Friard, O. and Gamba, M. (2016). BORIS: a free, versatile open-source event-logging software for video/audio coding and live observations. *Methods Ecol. Evol.* 7, 1325-1330. 10.1111/2041-210X.12584

[JEB249323C13] Günther, R. H., O'Connell-Rodwell, C. E. and Klemperer, S. L. (2004). Seismic waves from elephant vocalizations: a possible communication mode? *Geophys. Res. Lett.* 31, 1-4. 10.1029/2004GL019671

[JEB249323C14] Hebets, E. A., Elias, D. O., Mason, A. C., Miller, G. L. and Stratton, G. E. (2008). Substrate-dependent signalling success in the wolf spider, *Schizocosa retrorsa*. *Anim. Behav.* 75, 605-615. 10.1016/j.anbehav.2007.06.021

[JEB249323C15] Hill, P. S. M. (2001). Vibration and animal communication: a review. *Am. Zool.* 41, 1135-1142. 10.1668/0003-1569(2001)041[1135:VAACAR]2.0.CO;2

[JEB249323C16] Hill, P. S.M., (2008). *Vibrational Communication in Animals*. Harvard University Press.

[JEB249323C17] Hill, P. S. M. (2009). How do animals use substrate-borne vibrations as an information source? *Naturwissenschaften* 96, 1355-1371. 10.1007/s00114-009-0588-8 19593539

[JEB249323C18] Hill, P. S. M. and Wessel, A. (2016). Biotremology. *Curr. Biol.* 26, R187-R191. 10.1016/j.cub.2016.01.054 26954435

[JEB249323C19] Hill, P. S. M., Lakes-Harlan, R., Mazzoni, V., Narins, P. M., Virant-Doberlet, M. and Wessel, A. (2019). *Biotremology: Studying Vibrational Behavior*, 6th edn. Springer Nature.

[JEB249323C20] Ilton, M., Saad Bhamla, M., Ma, X., Cox, S. M., Fitchett, L. L., Kim, Y., Koh, J., Krishnamurthy, D., Kuo, C. Y., Temel, F. Z. et al. (2018). The principles of cascading power limits in small, fast biological and engineered systems. *Science* 360, eaao1082. 10.1126/science.aao1082 29700237

[JEB249323C21] Jordão, J. M., Curto, A. F. and Oliveira, R. F. (2007). Stereotypy and variation in the claw waving display of the fiddler crab *Uca tangeri*. *Acta Ethol.* 10, 55-62. 10.1007/s10211-007-0030-1

[JEB249323C22] Krebs, J. R. and Davies, N. B., (1978). *Behavioural Ecology: An Evolutionary Approach*. Wiley.

[JEB249323C23] Margerin, L., (2011). Seismic waves, scattering. In *Encyclopedia of Solid Earth Geophysics* (ed. H. K. Gupta), pp. 1210-1223. Dordrecht: Springer.

[JEB249323C24] Mason, M. J. and Narins, P. M. (2002). Seismic sensitivity in the desert golden mole (*Eremitalpa granti*): a review. *J. Comp. Psychol.* 116, 158-163. 10.1037/0735-7036.116.2.158 12083610

[JEB249323C25] Mitoyen, C., Quigley, C. and Fusani, L. (2019). Evolution and function of multimodal courtship displays. *Ethology* 125, 503-515. 10.1111/eth.12882 31341343 PMC6618153

[JEB249323C26] Mortimer, B. (2017). Biotremology: Do physical constraints limit the propagation of vibrational information? *Anim. Behav.* 130, 165-174. 10.1016/j.anbehav.2017.06.015

[JEB249323C27] Mortimer, B., Rees, W. L., Koelemeijer, P. and Nissen-Meyer, T. (2018). Classifying elephant behaviour through seismic vibrations. *Curr. Biol.* 28, R547-R548. 10.1016/j.cub.2018.03.062 29738725

[JEB249323C28] Mortimer, B., Walker, J. A., Lolchuragi, D. S., Reinwald, M., Daballen, D. and Mortimer, B. (2021). Noise matters: elephants show risk-avoidance behaviour in response to human-generated seismic cues. *R. Soc. Interface Proc. B* 288, 20210774. 10.1098/rspb.2021.0774 PMC8242925 34187196

[JEB249323C29] Mowles, S. L., Jennions, M. and Backwell, P. R. Y. (2017). Multimodal communication in courting fiddler crabs reveals male performance capacities. *R. Soc. Open Sci.* 4, 161093. 10.1098/rsos.161093 28405396 PMC5383853

[JEB249323C30] Mulder, T., Mortimer, B. and Vollrath, F. (2020). Functional flexibility in a spider's orb web. *J. Exp. Biol.* 223, jeb234070. 10.1242/jeb.234070 33184053

[JEB249323C31] O'Connell-Rodwell, C. E., Arnason, B. T. and Hart, L. A. (2000). Seismic properties of Asian elephant (*Elephas maximus*) vocalizations and locomotion. *J. Acoust. Soc. Am.* 108, 3066-3072. 10.1121/1.1323460 11144599

[JEB249323C32] O'Connell-Rodwell, C. E., Hart, L. A. and Arnason, B. T. (2001). Exploring the potential use of seismic waves as a communication channel by elephants and other large mammals. *Am. Zool.* 41, 1157-1170. 10.1093/icb/41.5.1157

[JEB249323C33] O'Connell-Rodwell, C. E., Wood, J. D., Kinzley, C., Rodwell, T. C., Poole, J. H. and Puria, S. (2007). Wild African elephants (*Loxodonta africana*) discriminate between familiar and unfamiliar conspecific seismic alarm calls. *J. Acoust. Soc. Am.* 122, 823-830. 10.1121/1.2747161 17672633

[JEB249323C34] Oliveira, R. F. and Custódio, M. R. (1998). Claw size, waving display and female choice in the European fiddler crab, *Uca tangeri*. *Ethol. Ecol. Evol.* 10, 241-251. 10.1080/08927014.1998.9522855

[JEB249323C35] Patek, S. N., Dudek, D. M. and Rosario, M. V. (2011). From bouncy legs to poisoned arrows: elastic movements in invertebrates. *J. Exp. Biol.* 214, 1973-1980. 10.1242/jeb.038596 21613512

[JEB249323C36] Randall, J. A. (1984). Territorial defense and advertisement by footdrumming in bannertail kangaroo rats (*Dipodomys spectabilis*) at high and low population densities. *Behav. Ecol. Sociobiol.* 16, 11-20. 10.1007/BF00293099

[JEB249323C37] Randall, J. A. (1997). Species-specific footdrumming in kangaroo rats: *Dipodomys ingens*, *D. deserti*, *D. spectabilis*. *Anim. Behav.* 54, 1167-1175. 10.1006/anbe.1997.0560 9398370

[JEB249323C38] Randall, J. A. and Boltas King, D. K. (2001). Assessment and defence of solitary kangaroo rats under risk of predation by snakes. *Anim. Behav.* 61, 579-587. 10.1006/anbe.2000.1643

[JEB249323C39] Randall, J. A. and Matocq, M. D. (1997). Why do kangaroo rats (*Dipodomys spectabilis*) footdrum at snakes? *Behav. Ecol.* 8, 404-413. 10.1093/beheco/8.4.404

[JEB249323C40] Reinwald, M., Moseley, B., Szenicer, A., Nissen-meyer, T., Oduor, S., Vollrath, F., Markham, A., Mortimer, B., Markham, A. and Seismic, M. B. (2021). Seismic localization of elephant rumbles as a monitoring approach. *R. Soc. Interface* 18, 1-11. 10.1098/rsif.2021.0264 PMC8277467 34255988

[JEB249323C41] Rivero, A., Alatalo, R. V., Kotiaho, J. S., Mappes, J. and Parri, S. (2000). Acoustic signalling in a wolf spider: Can signal characteristics predict male quality? *Anim. Behav.* 60, 187-194. 10.1006/anbe.2000.1452 10973720

[JEB249323C42] Rundus, A. S., Sullivan-Beckers, L., Wilgers, D. J. and Hebets, E. A. (2011). Females are choosier in the dark: Environment-dependent reliance on courtship components and its impact on fitness. *Evolution (N. Y)* 65, 268-282. 10.1111/j.1558-5646.2010.01125.x 20825477

[JEB249323C43] Salmon, M. and Atsaides, S. P. (1968). Visual and acoustical signalling during courtship by fiddler crabs (genus *Uca*). *Integr. Comp. Biol.* 8, 623-639. 10.1093/icb/8.3.623

[JEB249323C44] Salmon, M. and Atsaides, S. P. (1969). Sensitivity to substrate vibrations in the fiddler crab, *Uca pu**g**ilator* Bosc. *Anim. Behav.* 17, 68-76.

[JEB249323C45] Snieder, R. (1986). The influence of topography on the propagation and scattering of surface waves. *Phys. Earth Planet. Inter.* 44, 226-241. 10.1016/0031-9201(86)90072-5

[JEB249323C46] Szenicer, A., Reinwald, M., Moseley, B., Nissen-Meyer, T., Mutinda Muteti, Z., Oduor, S., McDermott-Roberts, A., Baydin, A. G. and Mortimer, B. (2021). Seismic savanna: machine learning for classifying wildlife and behaviours using ground-based vibration field recordings. *Remote Sens. Ecol. Conserv.* 8, 1-15. 10.1002/rse2.242

[JEB249323C47] Takeshita, F. and Murai, M. (2016). The vibrational signals that male fiddler crabs (*Uca lactea*) use to attract females into their burrows. *Sci. Nat.* 103, 49. 10.1007/s00114-016-1371-2 27240863

[JEB249323C48] Takeshita, F., Murai, M., Matsumasa, M. and Henmi, Y. (2018). Multimodal signaling in fiddler crab: waving to attract mates is condition-dependent but other sexual signals are not. *Behav. Ecol. Sociobiol.* 72, 140.

[JEB249323C49] Wolfrath, B. (1993). Observations on the behaviour of the European fiddler crab *Uca tangeri*. *Mar. Ecol. Prog. Ser.* 100, 111-118. 10.3354/meps100111

[JEB249323C50] Žunič, A., Cokl, A., Doberlet, M. V. and Millar, J. G. (2008). Communication with signals produced by abdominal vibration, tremulation, and percussion in *Podisus maculiventris* (Heteroptera: Pentatomidae). *Ann. Entomol. Soc. Am.* 101, 1169-1178. 10.1603/0013-8746-101.6.1169

