# Supplementary material for: Constraints on percussive seismic signals in a noisy environment by European fiddler crabs, Afruca tangeri
Source: J Exp Biol. 2025 Apr 10;228(7):jeb249323. doi: 10.1242/jeb.249323 (PMC12045639; doi:10.1242/jeb.249323)
Supplement: Supplementary information [file jexbio-228-249323-s1.pdf]

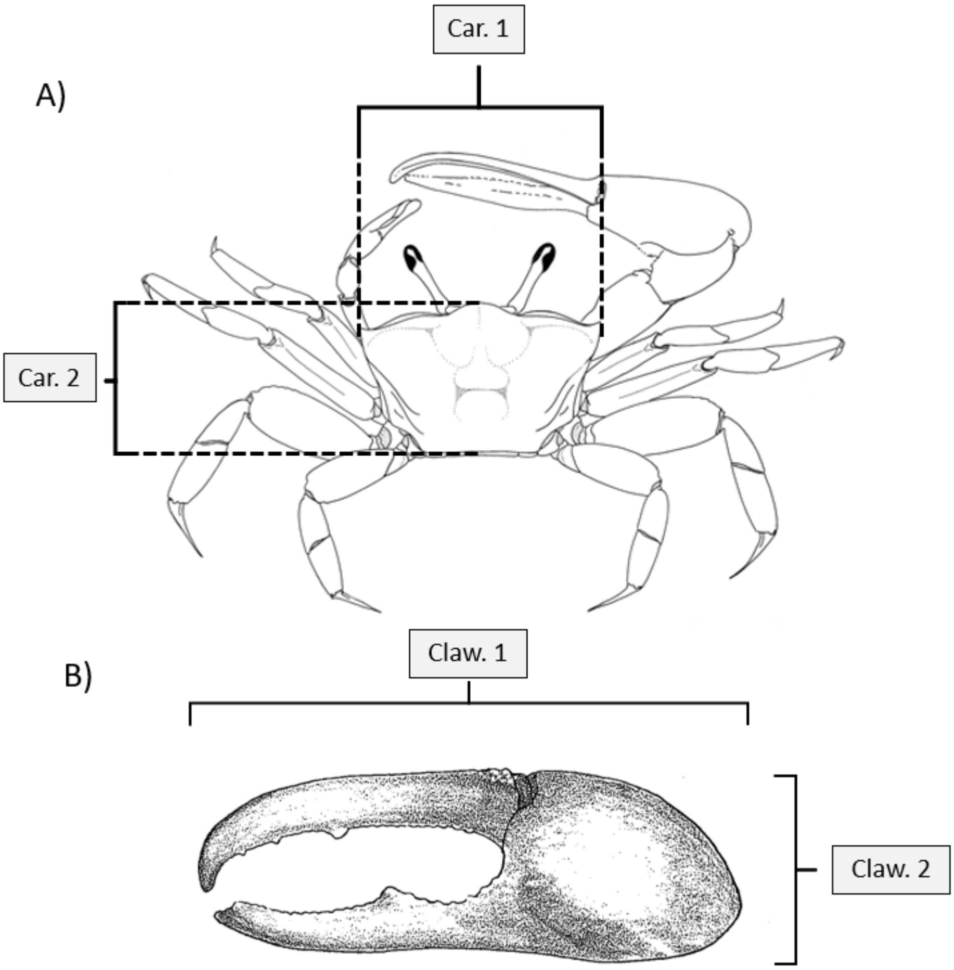

Fig. S1. Male *Uca sp.* diagram indicating measured features (modified from (Crane, 1975; Rosenberg, 2023)). Car.1 = Carapace width, Car.2 = Carapace length, Claw.1 = Claw length, Claw.2 = Claw width. Modifications of the original image include the brackets indicating the measured components.

Journal of Experimental Biology • Supplementary information

Table S1. Ethogram of behaviours observed in video recordings.

Behaviour	Type	Description	No. retained seismic recordings*
Waving	Point	Distal end of the major claw is extended outwards from the body on the side of the major claw, raised up in an arc, and lowered down rapidly in front of the body. The body of the crab remains stationary throughout.<br>Claw waving motion much like the lateral wave described in (see (How et al., 2008)).<br>**Behaviour always tagged at the end of the movement (± 0.2 s).**	*n* = 3986
Simultaneous wave and body drop<br><br>(body drop component aka, "body thumping")	Point	The major claw is raised directly up above the body whilst the body is raised up by extension of the walking legs, and subsequently the body and claw are simultaneously and rapidly dropped down to the original lowered position with a jerky motion. There appears to be carapace-sand contact, and claw-sand contact is common.<br>Claw waving motion like the vertical wave described in (see (How et al., 2008; Jordão et al., 2007)).<br>**Behaviour always tagged at the end of the movement (± 0.2 s).**	*n* = 269
Sequential wave and body drop:		The distal end of the major claw is extended outwards from the body on the side of the major claw and raised up in an arc whilst the body is simultaneously raised by extension of the walking legs.	*n seqW* = 1239<br>*n seqBD* = 1249
seqW	Point	Accordingly, the claw is dropped down to its original position in front of the body. Claw waving motion much like the lateral wave described in(How et al., 2008; Jordão et al., 2007). There are occasions of claw-sand contact at the end of the motion, but this appears incidental.	
seqBD	Point	**Behaviour always tagged at the end of the waving movement (± 0.2 s).**<br><br>Once the claw is lowered, the body is dropped down to its original lowered position with a jerky motion. There appears to be	

Journal of Experimental Biology • Supplementary information

		carapace-sand contact at the end of the motion. **Behaviour always tagged at the end of the movement (± 0.2 s).**	
Locomotion	State	The crab moves across the substrate using its walking legs. **Tag starts when behaviour is initiated and stops when behaviour is terminated (± 0.2 s).**	*n* = 626
Mudballing	State	The crab cups a mud ball using two or more walking legs and drags it from inside the burrow to a collection point of mud balls outside the burrow. This behaviour was tagged from the moment the crab exited the burrow to when the mudball was deposited. If the crab walked back to the burrow, the return component was tagged as locomotion. **Tag starts when crab exits the burrow with mudball and stops when the mudball has been released (± 0.2 s).**	*n* = 383
Conflict	State	A non-focal male or female passes near the focal burrow of the focal male raises its major claws in front of its body whilst non-focal crab. Occasionally conflicts escalated where the major claws of males were interlocked for a fight. Conflicts sometimes resulted in the non-focal male taking over occupancy of the focal burrow. **Tag starts when the claw of the focal male is first raised and stops when one of the crabs leaves the area or the claw was lowered (± 0.2 s)**	*n* = 125
Aboveground drumming	State	The crab makes a drumming-like motion with the base of its claw and a drumming sound is heard. No contact is observed between the claw and the substrate. Where stints of drumming are interluded by substantial quiet moments these are denoted as separate drumming bouts/events. **Tag starts when behaviour is initiated and stops when behaviour is terminated (± 0.2 s).**	*n* = 111
Underground drumming	State	A drumming sound is heard whilst the focal crab is inside its burrow. Where stints of drumming are interluded by substantial quiet	*n* = 219

Journal of Experimental Biology • Supplementary information

		moments these are denoted as separate drumming bouts/events.<br>Tag starts when behaviour is first heard in the audio of the video recording, and stops when drumming sound is no longer heard (± 0.6 s).	
*Seismic recordings of behaviour were retained or excluded from the behaviour catalogue based on parameters described in Methods. seqW and seqBD always occurred as a paired behaviour.			

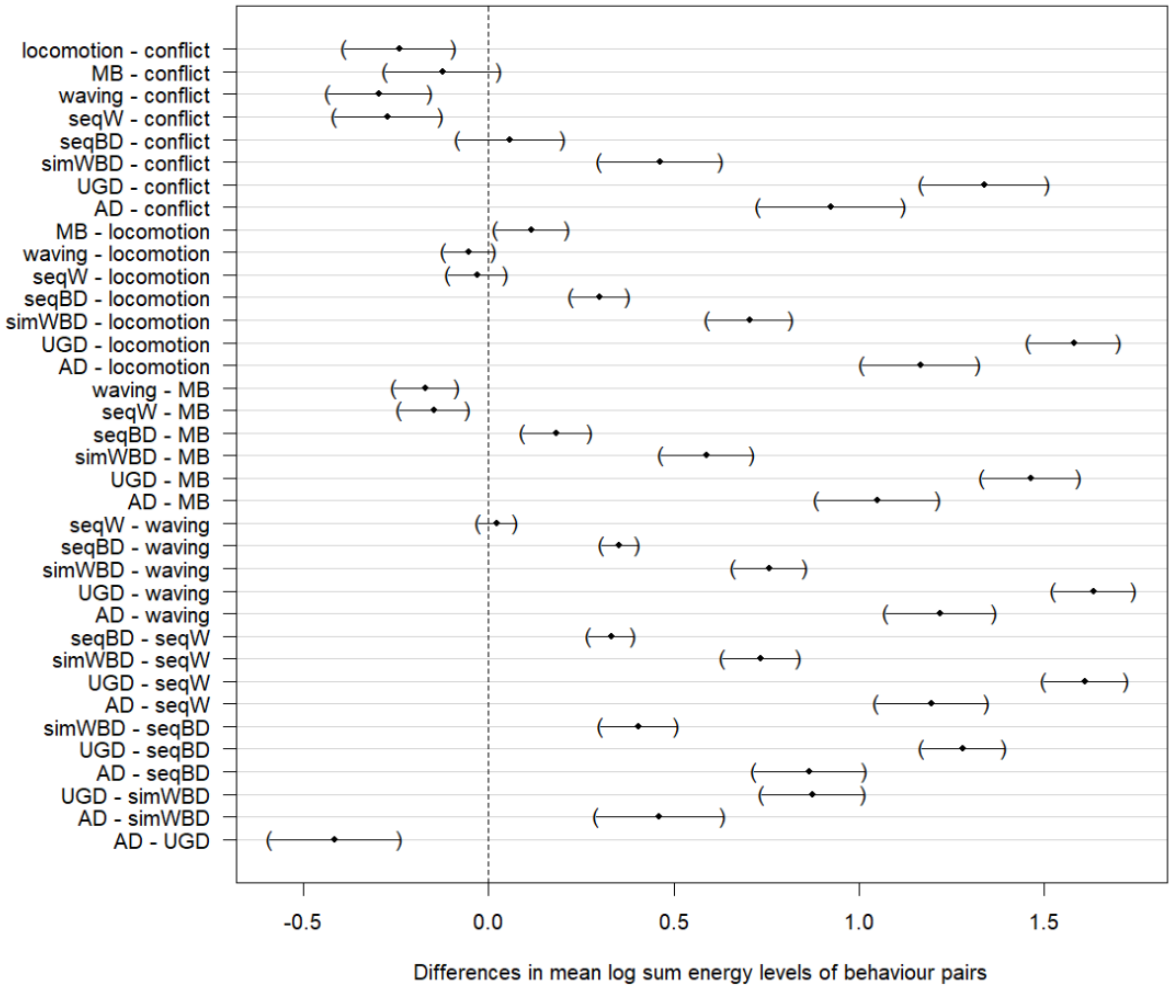

Fig. S2. Pairwise comparisons of energy levels of individual behaviours. Tukey test pairwise comparison results on LMM_{LE} visualised with 95% family-wise confidence intervals. Where error bars of compared pair overlap with 0 (vertical dashed line), difference is non-significant ($p > 0.05$).

Journal of Experimental Biology • Supplementary information

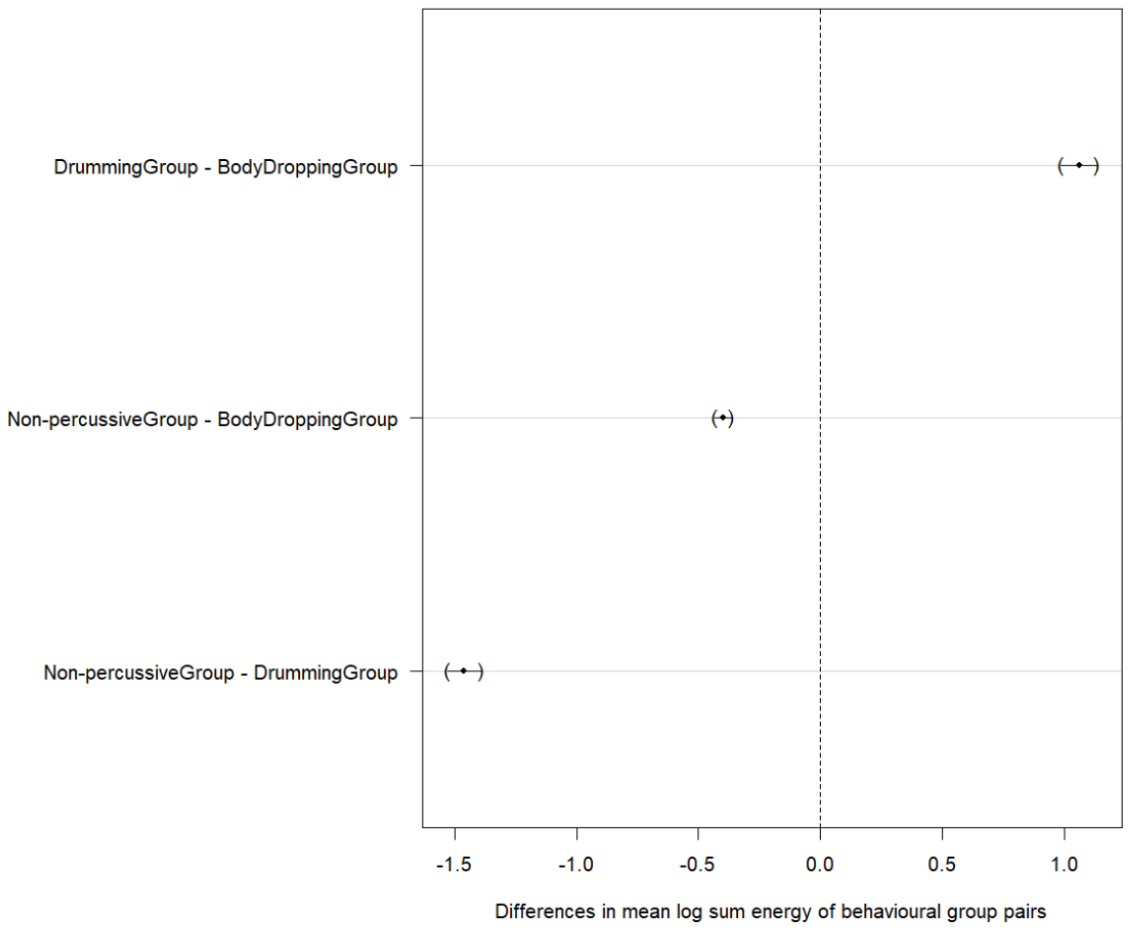

Fig. S3. Pairwise comparisons of energy levels of behavioural groups. Tukey test pairwise comparison results visualised with 95% family-wise confidence intervals. Where error bars overlap with 0 (vertical dashed line), difference is non-significant ($p > 0.5$). Non-percussive group: conflict, locomotion, MB, waving and seqW. Body dropping group: simWBD and seqBD. Drumming group: AD, UGD behaviours. See Supplementary methods Table 2 for detailed group descriptions.

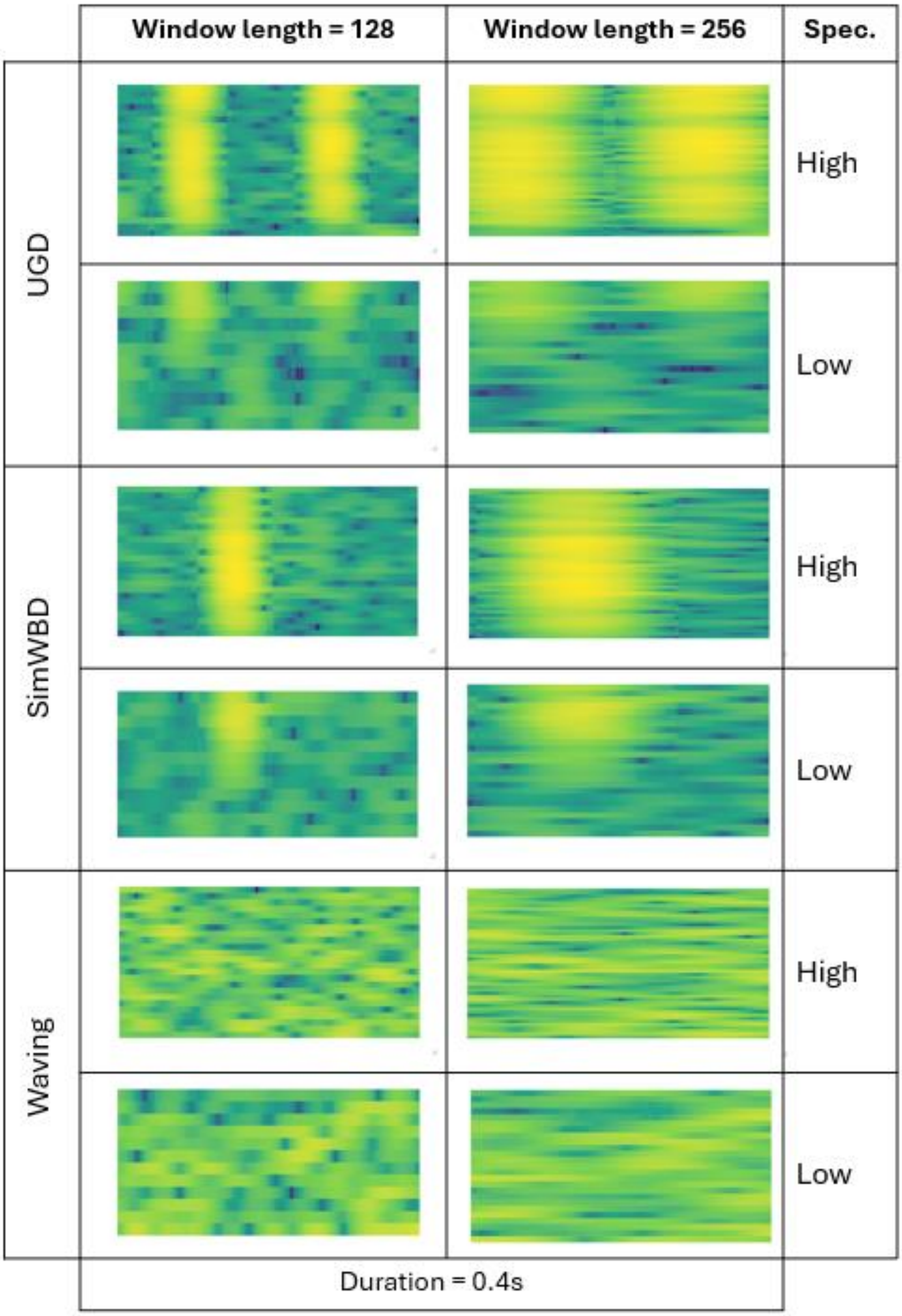

Fig. S4. Spectrograms associated with exemplar behaviours in Fig. 4 of main text. Frequency range for the Low frequency spectrograms = 4.5 - 160 Hz, and for High frequency spectrograms 100-400 Hz. Left column indicates spectrograms with window length = 128 samples, and right column indicates spectrograms with window length = 256 samples. All spectrograms are 0.4s in duration.

Table S2. Sum energy = energy Ch0 + energy Ch2, R^2 Conditional = variance captured by fixed + random effect variables, R^2 Marginal = variance captured by fixed effect variables, T values for LMMs, Z value for GLMMs, where models are considered families rank is determined by ascending P-value in family of tests. B-H critical value = (rank / no. tests) × false discovery rate. False discovery rate = 0.1. * = significant effect controlling for false discovery rate using Benjamini–Hochberg procedure. Sections each refer to model family groups or collections of models.

Section A)
Effect of morphology on drumming signal features - interaction models (model family A)

Model name	Rank	Lowest interaction P value	B-H critical value	t value	R^2 Marginal	R^2 Conditional
LMM_{LEClaw}	1	4.92×10^{-12}*	0.025	6.93	0.26	0.57
LMM_{LECar}	2	3.8×10^{-6}*	0.050	4.63	0.24	0.59
LMM_{FCar}	4	0.19	0.075	1.32	0.02	0.18
LMM_{FClaw}	3	0.21	0.100	1.25	0.03	0.18

Section B)
Effect of morphology on energy of seismic signals - post-hoc models (model family B)

Behaviour Group	Model name	Rank	P value	B-H critical value	t value	R^2 Marginal	R^2 Conditional
Drumming	$LMMD_{EnergyClaw}$	1	0.016*	0.017	2.63	0.08	0.26
Drumming	$LMMD_{EnergyCar}$	2	0.223	0.033	1.25	0.02	0.28
Body dropping	$LMMBD_{EnergyClaw}$	3	0.259	0.05	-1.15	0.01	0.25
Body dropping	$LMMBD_{EnergyCar}$	4	0.34	0.067	-0.97	0.01	0.25
Non-percussive	$LMMNP_{EnergyClaw}$	5	0.44	0.083	-0.79	0.01	0.48
Non-percussive	$LMMNP_{EnergyCar}$	6	0.48	0.1	0.72	0.01	0.48

Section C)
Effect of morphology on drumming spikes (model family C)

Model name	Rank	P value	B-H critical value	T / Z value	R^2 Marginal	R^2 Conditional
$LMM_{AmpDrum}$	1	0.011*	0.033	2.81	0.09	0.45
$GLMM_{BoutsizeDrum}$	2	0.136	0.067	-1.49	0.05	0.54
$LMM_{\Delta tDrum}$	3	0.545	0.1	0.62	0.01	0.64

Section D)
Effect of environmental variables on seismic background noise.

Model name	Fixed effect variable(s)	T value	P value	R^2 Marginal	R^2 Conditional
$LMM_{NoiseInteraction}$	Vegetation dist.<br>Wind speed<br>Wind x veg. dist.	1.64<br>7.84<br>-0.54	0.10<br><0.001*<br>0.59	0.33	0.67
LMM_{Noise}	Vegetation dist.<br>Wind speed	1.96<br>9.87	0.06<br><0.001*	0.33	0.66
$LMM_{WindNoise}$	Wind speed	10.11	<0.001*	0.26	0.65

Section E)
Effect of crab morphology on distance to vegetation (model family E)

Model name	Rank	P value	B-H critical value	T value	R^2 Marginal	R^2 Conditional
$LMM_{PositionFemales}$	1	0.18	0.05	-1.40	0.09	0.30
$LMM_{PositionMales}$	2	0.56	0.1	-0.59	0.005	0.47

Supplementary Materials and Methods

Erroneous recordings

Removed examples include: first, instances where signal amplitude was substantially greater in the far geophone pair than the close geophone pair. Here it could be safely assumed that the spikes were produced by a non-focal crab nearer to the far geophone pair than the close geophone pair. Second, if an extensive multi-spike pattern was observed for a point behaviour with a singular movement like waving, it was removed. Here it could be assumed the geophone captured a drumming behaviour by a non-focal crab. Third, multi-bout drumming events where 3 or more bouts were captured as one drumming event were removed. Such errors occurred because underground drumming was more difficult to establish (based on audio), and the observer occasionally accidentally captured multiple drumming bouts as a singular drumming bout.

Machine Learning

In detail, we employed the negative log-likelihood loss function alongside the Adam optimizer (Kingma and Ba, 2014) to monitor and adjust the validation set loss during hyperparameter tuning. We initiated the learning rate at 0.001, with a strategic adjustment to reduce it to 95% of its preceding value after each set of 5 epochs. This approach was facilitated by utilizing a single NVIDIA A10 GPU coupled with 24 GB of RAM to support both training and validation processes.

We used 10-fold cross-validation to estimate the skill of a machine learning model. This involves splitting the entire dataset into 10 equal parts, or 'folds.' The model is then trained on 9 folds and tested on the remaining 1 fold. This process is repeated 10 times, with each fold being used as the test set once. The final performance of the model is determined by averaging the performance across all 10 tests. This technique helps to ensure that the model's performance is reliable and not dependent on a particular way the data is split (Wong, 2015).

The ResNet model was previously trained on a large dataset, typically on a wide-ranging task that is not too specific. These models are used as starting points for various machine learning and deep learning tasks and can be fine-tuned or adapted to perform different, often more specific, tasks than the original training task. This approach is beneficial because it allows for the transfer of knowledge from one domain to another, often leading to improved performance when data for the specific task is limited. Here, we use a typical computer vision-based pre-trained model-ResNet (He, 2016) for initial feature extraction, which will reduce the number of training data and speed up the model convergence.

Data augmentation was used to increase the diversity of our training data without actually collecting new data. This is done by applying various transformations to the existing data, such as rotating, adding noise, or randomly altering the syntax of images. These transformations create additional training examples that are similar but not identical to the original data, which can help improve the robustness and generalization ability of a machine-learning model. This is especially useful in situations where data is scarce or expensive to collect.

In our specific neural network, we additionally used batch normalization (Sergey and Szegedy, 2015) and dropout (Nitish et al., 2014) to yield a more robust ML model. Batch normalization is applied to stabilize and accelerate training by normalizing the input layer by re-centering and re-scaling. It works by adjusting and scaling the activations of each layer's inputs over a mini-batch, thus helping to mitigate the problem known as "internal covariate shift." Dropout is a regularization technique used in neural networks to prevent overfitting by randomly dropping units and their connections during training, which forces the network to learn more robust features. This method effectively creates a "thinned" version of the network at each iteration, leading to better generalization when making predictions on new data.

Statistics

Seismic signal differences between individual behaviours

The sum energy variable from the seismic data correlated strongly with the amplitude variables (correlation value ≥ 0.70). Sum energy was thus deemed a suitable representative of the peak amplitude variables, which therefore were not tested. Peak frequency did not correlate strongly with any of the other variables (max. correlation value = 0.16). The difference in peak frequency and sum energy as a function of behaviour ($n_{behaviours}$ = 9, table 1) was subsequently tested with two linear mixed models and associated Tukey pairwise tests.

The peak frequency (LMM_F) and log of energy (LMM_{LE}) were the dependent variables in each model respectively, while behaviour was the independent variable and focal burrow ID was the random effect variable in both models. Energy was log transformed to yield an approximately normal distribution of residuals for LMM_{LE} but residuals were under-dispersed for LMM_{LE}. Dependent variable transformation was attempted for sum energy based on the Cox-Box Radar plot, but this resulted in a failure to converge. Hence the model with the log-transformed sum energy variable was used going forward despite under-dispersed residuals; yielding conservative results.

R^2 values of the models indicate that focal burrow ID, explained a substantial amount of variance (LMM_{LE}: 13% and LMM_F: 15%) and thus had to be retained. However, where the fixed effect variable (behaviour) also explains 27% of the variance in LMM_{LE}, behaviour only explained 2% of variance in LMM_F. Frequency was hence deemed uninformative for the purpose of distinguishing behaviours from signal features, and frequency differences between behaviours were not explored further. A Tukey test was applied to LMM_{LE} for pairwise comparisons of log sum energy values of the 9 behaviours.

Signal differences between behavioural groups

An additional test was performed with the same model structure as LMM_{LE} where the independent fixed effect variable was changed from "Behaviour" to "Behavioural Group" ($LMMG_{LE}$). The residuals were approximately normally distributed, albeit again with some under-dispersion. Transformation per the Box-Cox Radar plot again resulted in failure to converge and the log-transformed sum energy variable was used going forward despite under-dispersed residuals, yielding conservative results.

R^2 values $LMMG_{LE}$ indicated that the random effect (burrow ID) and fixed effect variable (behavioural group) both explained a substantial amount of variance (13% and 25% respectively). A Tukey test was hence applied to $LMMG_{LE}$ for pairwise comparisons of log energy between the 3 behavioural groups.

Signaller morphology and seismic signal features

The effect of claw size and carapace size on peak frequency ($LMM_{FClaw,}$ LMM_{FCar}) and log of the sum energy ($LMM_{LEClaw,}$ LMM_{LECar}) were tested with four linear mixed models with an interaction. In these models, peak frequency or the log of the sum energy were the dependent variables, claw or carapace size and behavioural group, as well as the interaction term between claw or carapace size and behavioural group, were the fixed effect variables. Crab ID was included as a random effect variable. The residuals were all approximately normally distributed for the frequency models, but under-dispersed for the log energy models. Transformation was attempted based on the Box-Cox Radar plot but again resulted in failure to converge. The log-transformed dependent variable was used for the energy models going forward, yielding conservative results. The false discovery rate was controlled for using the Benjamen-Hochberg procedure (Benjamini and Hochberg, 1995), assuming a 0.1 false discovery rate across this family of 4 tests (model family A).

Adjusted R^2 values indicated that the fixed effect variables only explained 2-3% of the variance in the frequency models ($LMM_{FClaw,}$ LMM_{FCar}) compared to 24% and 26% in the log energy models (LMM_{LEClaw}, LMM_{LECar} respectively). The effect of morphology on peak frequency was hence not explored further. For both energy models, the interaction term was highly significant (where $p < 0.001$, Supplementary Results Table 1A). As such, six individual post-hoc energy models further tested the effect of morphology (carapace size and claw size) on sum energy for each of the three behavioural groups.

The six post-hoc energy models initially included the log of sum energy as the dependent variable, the morphological measure (claw size or carapace size) as the independent variable, and Crab ID as the random effect variable to account for repeated measures. Although the residuals of the drumming group models were normally distributed, the residuals of the body dropping, and non-percussive

group models were severely right-tailed and under-dispersed despite the initial log transformation. A Cox-Box Radar plot showed that 1/sum energy and 1/sum energy^2 transformed dependent variables were more suitable for the drumming and non-percussive models respectively- yielding normally distributed residuals. The false discovery rate was controlled for using the Benjamen-Hochberg procedure (Benjamini and Hochberg, 1995), assuming a 10% false discovery rate across this family of six tests (model family B). The random effect variable explained substantial a substantial amount of variance (≥ 18%) and was thus important to retain in all models.

Signaller morphology and drumming peaks

Only the effect of claw size on drumming bout peaks was explored further, because claw size was the only morphological feature with a significant effect on seismic drumming energy ($LMMD_{EnergyClaw}$, Supplementary Results Table 1B). To assess the effect of claw size on the absolute amplitude of drumming amplitude peaks, drumming rate and bout size (= no. peaks), the dataset was firstly filtered to retain examples that were correctly assigned to the "Drumming group" by the ML algorithm (*n* = 221) and had associated morphological crab measurements. In total 161 drumming bouts were correctly identified and associated with 24 measured males.

Drumming peaks were identified as absolute amplitude peaks in channel 2 that exceeded 1/4 of the maximum absolute amplitude reading in the given bout and exceeded the absolute amplitude of the preceding and following 100 readings (i.e. 0.062 s). The effect of claw size on absolute peak amplitude was explored using all identified drumming peaks (*n* = 1397). Drumming rate (= Δt between absolute peaks in a singular bout) was calculated for all peaks except the first peak in a bout. Where Δt > 0.3s occurred in a recording, multiple sequential bouts were accidentally captured as a singular drumming event – causing this uncharacteristically large Δt value. In case of this BORIS tagging error, the bout with the most peaks was retained for each tagged event, yielding *n* = 1350 Δt values.

The effect of claw size on absolute drum amplitude and drumming rate was tested using two LMMs ($LMM_{AmpDrum}$, $LMM_{\Delta tDrum}$), with log absolute peak amplitude and Δt as the fixed effect variables, and crab ID as the random effect variable to adjust for repeated measures. Residuals of both models were approximately normally distributed and met assumptions. The effect of claw size on bout size was tested with a Poisson Generalised Linear Mixed Model ($GLMM_{BoutsizeDrum}$) to analyse the count-based dependent variable, claw size as the fixed effect variable, and crab ID as the random effect variable to adjust for repeated measures. The false discovery rate was controlled for using the Benjamen-Hochberg procedure (Benjamini and Hochberg, 1995), assuming a 10% false discovery rate across this family of three tests (model family C).

Drumming peak attenuation

For drumming behaviour, we further explored how peak frequency and signal energy was affected by distance propagated, and if claw size (Claw.1 × Claw.2, Figure 3 B) affected this change. Two linear mixed models with an interaction term ($LMM_{FDistance}$, $LMM_{LEDistance}$) tested the effect of distance to geophone on peak frequency and log of the sum energy of recorded by channels 1 and 3 (distance: 20-100cm, Manuscript Figure 1). In these models, peak frequency or the log of the sum energy were the dependent variables, while claw size and distance from the far geophone pair to the burrow, as well as their interaction term, were the fixed effect variables. Burrow ID was included as a random effect variable to control for repeated measures. The residuals were normally distributed for both models. The false discovery rate was controlled for using the Benjamen-Hochberg procedure

Journal of Experimental Biology • Supplementary information

(Benjamini and Hochberg, 1995), assuming a 0.1 false discovery rate across this family of two tests (model family D).

The fixed effect variables in $LMM_{FDistance}$ explained less than 1% of the variance, and distance effects on frequency were hence not explored further. In the energy model ($LMM_{LEDistance}$) the interaction term was found to be non-significant, but the fixed effects explained a substantial amount of variance (17%). For the final model ($LMMLE_{DistanceFinal}$) the interactive term was removed per the stepwise regression method because it was not significant, and claw size was removed because the independent effect of claw size on signal energy has already been tested in the previous section. The residuals of the final model were normally distributed, while the fixed and random effect variables both explained a substantial amount of variance (13% and 36% respectively) and were thus both important to retain.

Environmental variables and seismic background noise

In the first and most complex model ($LMM_{NoiseInteraction}$), log of the seismic energy was included as the dependent variable, while wind speed and distance to vegetation as well as their interaction term were included as the fixed effect variables. Burrow ID was included as a random effect variable to adjust for repeated measures. The residuals of $LMM_{NoiseInteraction}$ were normally distributed and model assumptions were met.

The interaction term in $LMM_{NoiseInteraction}$ did not have a significant effect on seismic noise (t-value =-0.54, p = 0.58). Per the stepwise regression method, the interaction variable was removed, and the fixed variables were only included separately, before the model was re-run ($LMM_{Noise,}$ Table 10). The residuals of LMM_{Noise} were normally distributed and model assumptions were met.

The vegetation distance variable in LMM_{Noise} did not have a significant effect on seismic noise (t = 1.96, p = 0.06), and was removed to yield the simplest model, $LMM_{WindNoise}$. The residuals of $LMM_{WindNoise}$ were normally distributed and assumptions were met.

Morphological variation on shoreline

To determine if crab morphology affects their vertical position on the shoreline, claw size of males and carapace size of females were correlated with distance to vegetation (which occurred only on the upper shore). Given the absence of large claws on females, carapace size was correlated with distance to vegetation ($LMM_{PositionFemales}$). For males, claw size was correlated to distance to vegetation ($LMM_{PositionMales}$) because it was the only morphological variable with a significant effect on signal features. In these models, distance to vegetation was included as the dependent variable, the morphological measures (claw size and carapace size respectively) were included as fixed effect variables, and transect ID was included as the random effect variable to adjust for non-independence of samples. Residuals were normally distributed based on visual inspection of the histograms of residuals. The false discovery rate was controlled for using the Benjamen-Hochberg procedure (Benjamini and Hochberg, 1995), assuming a 10% false discovery rate across this family of two tests (model family E). Inclusion of the random effect variable was important as it explained a substantial amount of variance in both models (> 20 %).

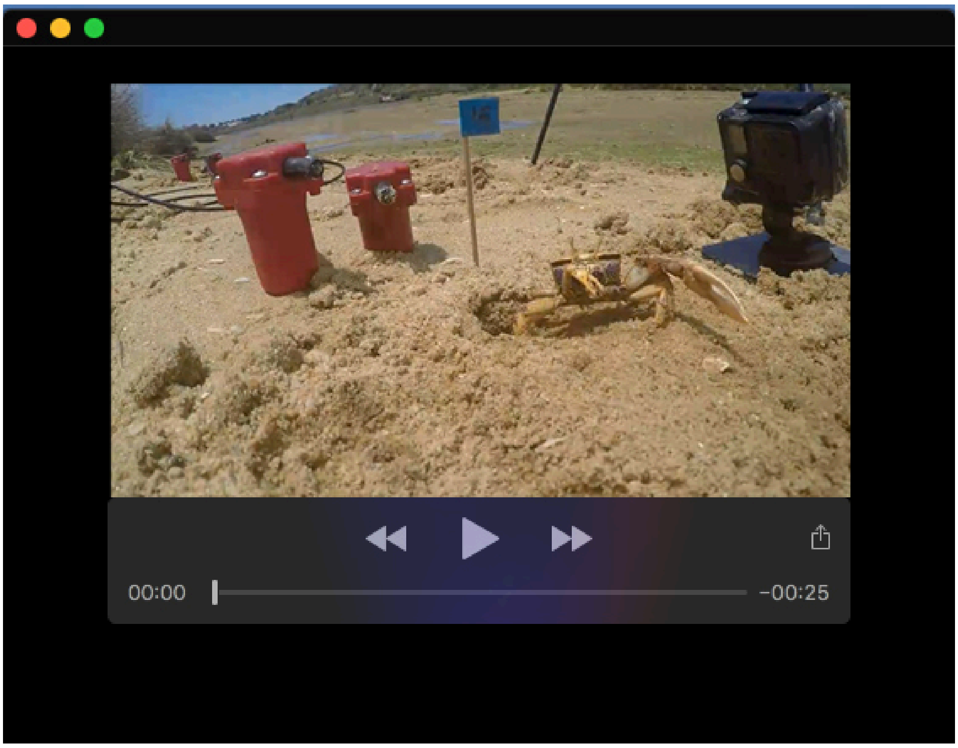

Movie 1. *Afruca tangeri* male performing the four-step courtship routine. Performing (1) the waving behaviour, followed by (2) sequential waves and body drops, (3) simultaneous waves and body drops and finally (4) underground drumming.

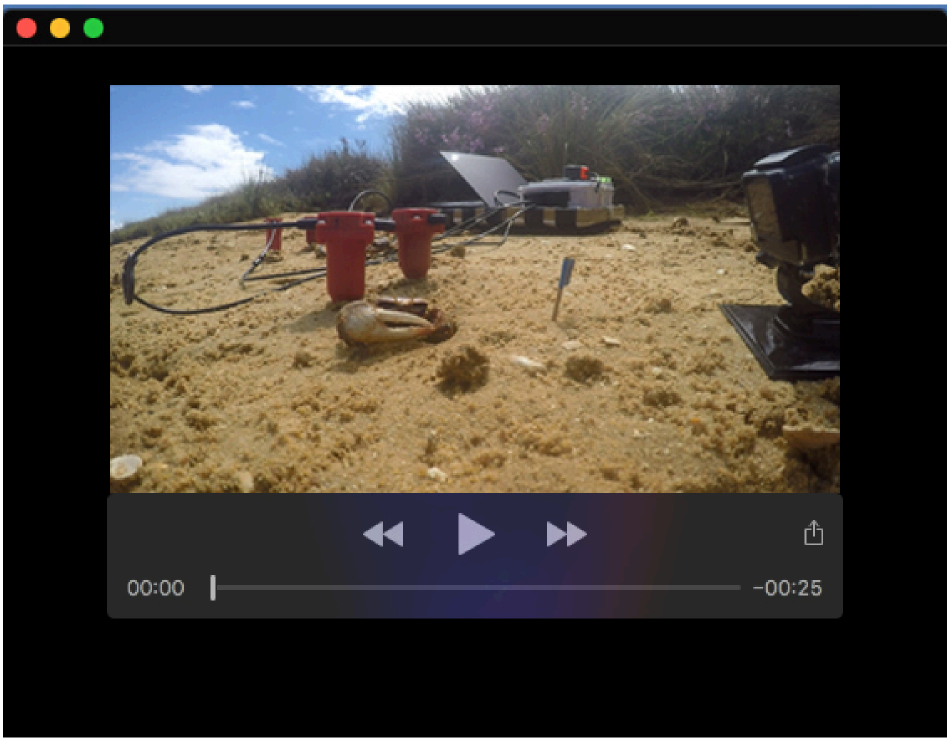

Movie 2. *Afruca tangeri* male performing aboveground drumming behaviour.

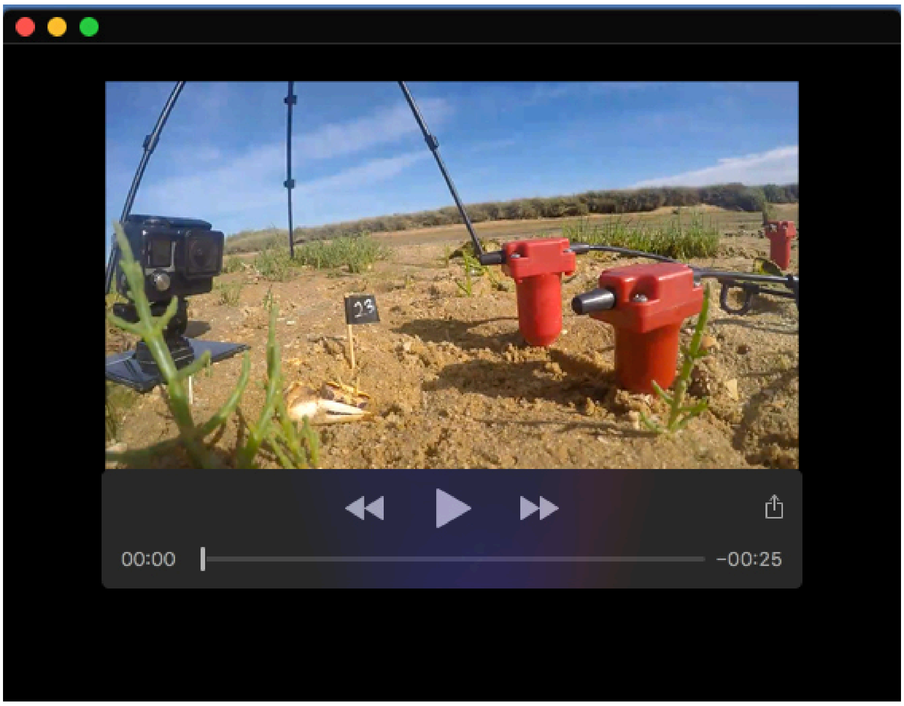

Movie 3. *Afruca tangeri* male performing the mudballing behaviour followed by locomotion back to the burrow entrance.

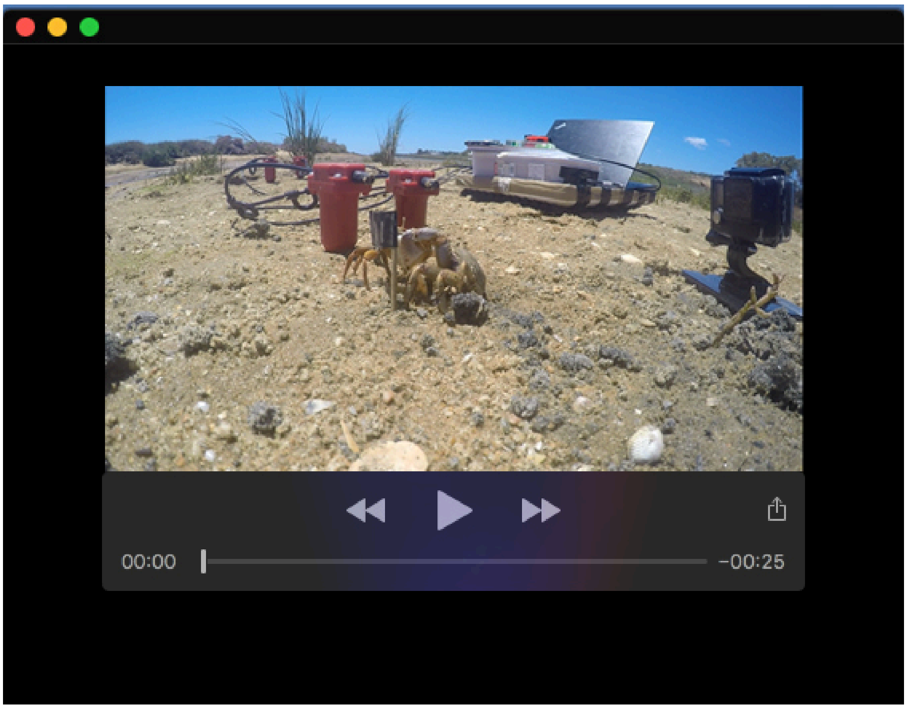

Movie 4. *Afruca tangeri* male performing the conflict behaviour towards another male.

Bibliography

Benjamini, Y., Hochberg, Y., 1995. Controlling the false discovery rate: a practical and powerful approach to multiple testing. *J. R. Stat. Soc. B* 57, 289–300.

Crane, J., 1975. Fiddler Crabs of the World.

He, K., 2016. Deep residual learning for image recognition, in: Proceedings of the IEEE Conference on Computer Vision and Pattern Recognition.

How, M.J., Hemmi, J.M., Zeil, J., Peters, R., 2008. Claw waving display changes with receiver distance in fiddler crabs, Uca perplexa. *Anim. Behav.* 75, 1015–1022. doi:10.1016/j.anbehav.2007.09.004

Jordão, J.M., Curto, A.F., Oliveira, R.F., 2007. Stereotypy and variation in the claw waving display of the fiddler crab Uca tangeri. *Acta Ethol.* 10, 55–62. doi:10.1007/s10211-007-0030-1

Kingma, D.P., Ba, J., 2014. Adam: A method for stochastic optimization. *arXiv* 1412.

Nitish, S., Hinton, G., Krizhevsky, A., Sutskever, I., Salakhutdinov, R., 2014. Dropout: a simple way to prevent neural networks from overfitting. *J. Mach. Learn. Res.* 15.

Rosenberg, M., 2023. fiddlercrab.info [WWW Document].

Sergey, I., Szegedy, C., 2015. Batch normalisation: Accelerating deep network training by reducing internal covariate shift, in: International Conference on Machine Learning.

Wong, T.T., 2015. Performance evaluation of classification algorithms by k-fold and leave-one-out cross validation. *Pattern Recognit.* 48.
